# Supplementary material for: Gene Regulatory Logic for Reading the Sonic Hedgehog Signaling Gradient in the Vertebrate Neural Tube
Source: Cell. 2012 Jan 20;148(1-2):273–84. doi: 10.1016/j.cell.2011.10.047 (PMC3267043; doi:10.1016/j.cell.2011.10.047)
Supplement: Document S1. Tables S1–S3 [file mmc1.pdf]

Table S1. Standardized Nomenclature for Mouse Embryo Staging, Related to Figure 1

Neural tube time (hph; hours post headfold stage)	Embryonic day	Number of somites
0hph	E7.5	0 (headfold stage)
8hph	E8.0	3-4
18hph	E8.4	8-9
20hph	E8.5	10-11
40hph	E8.75	20-22
50hph	E9.0	25-27
60hph	E9.5	30-32
70hph	E10.0	35-37
80hph	E10.5	40
90hph	E11.0	NA
100hph	E11.5	NA

Table S2. Set of Parameter Values Generating the Relevant Biological Outputs, Related to Figure 4

(Initial value for P =3)

Parameters	Description	Value
α	Maximum rate of P	3
β	Maximum rate of O	5
γ	Maximum rate of N	5
h_1	Strength of N repression on P	6
h_2	Strength of O repression on P	2
h_3	Strength of N repression on O	5
h_4	Strength of O repression on N	1
h_5	Strength of P repression on N	1
k_1	Rate of P degradation	1
k_2	Rate of O degradation	1
k_3	Rate of N degradation	1
O_{critP}	Critical value of O for which P value has reached half of its maximal	1
N_{critP}	Critical value of N for which P value has reached half of its maximal	1
O_{critN}	Critical value of O for which N value has reached half of its maximal	1
N_{critO}	Critical value of N for which O value has reached half of its maximal	1
P_{critN}	Critical value of P for which N value has reached half of its maximal	1
n	Hill coefficient of G cooperativity on O	1
m	Hill coefficient of G cooperativity on N	1

Table S3. Identification of the Parameters Robust or Sensitive to a 2-Fold Increase or Decrease in Their Values, Related to Figure 4

The tick marks indicate the parameter changes for which the system adopts the biological relevant outputs over time or when G is increased, as well as in conditions where P or O were removed (α or β=0, respectively). A, B, C, D indicate that the system fails to adopt the biological relevant output when the parameter is changed. In failure A, the system adopts an O^{HIGH} state for all values of G above a certain threshold and N is never induced. B indicates that in the absence of O, P prevails for all values of G and N is never induced. P, O and N coexist even at high values of G in Failure C. In Failure D, O is not induced for any value of G.

**Parameters**	***Value doubled***	***Value halved***
α	✓	✓
β	*A*	✓
γ	✓	*A,B*
h_1	✓	✓
h_2	✓	✓
h_3	✓	✓
h_4	*A*	✓
h_5	*B*	✓
k_1	✓	*B*
k_2	✓	*A*
k_3	*A,B*	✓
N_{critP}	*C,B*	✓
O_{critP}	✓	✓
N_{critO}	*A*	*D*
O_{critN}	✓	*A*
P_{critN}	✓	✓
n	✓	✓
m	✓	✓
