# Supplementary material for: Gene Regulatory Logic for Reading the Sonic Hedgehog Signaling Gradient in the Vertebrate Neural Tube
Source: Cell. 2012 Jan 20;148(1-2):273–84. doi: 10.1016/j.cell.2011.10.047 (PMC3267043; doi:10.1016/j.cell.2011.10.047)
Supplement: Document S1. Article plus Supplemental Information [file mmc2.pdf]

Cell

Gene Regulatory Logic for Reading the Sonic Hedgehog Signaling Gradient in the Vertebrate Neural Tube

Nikolaos Balaskas,[1,3,4] Ana Ribeiro,[1,3] Jasmina Panovska,[2,3] Eric Dessaud,[1,3] Noriaki Sasai,[1] Karen M. Page,[2] James Briscoe,[1,*] and Vanessa Ribes[1]
[1]Developmental Biology, National Institute for Medical Research, Mill Hill, London NW7 1AA, UK
[2]Department of Mathematics, University College London, Gower Street, London WC1E 6BT, UK
[3]These authors contributed equally to this work
[4]Present address: Department of Biochemistry and Molecular Biophysics, Columbia University Medical Center, New York, NY 10032, USA
*Correspondence: james.briscoe@nimr.mrc.ac.uk
DOI 10.1016/j.cell.2011.10.047

SUMMARY

Secreted signals, known as morphogens, provide the positional information that organizes gene expression and cellular differentiation in many developing tissues. In the vertebrate neural tube, Sonic Hedgehog (Shh) acts as a morphogen to control the pattern of neuronal subtype specification. Using an in vivo reporter of Shh signaling, mouse genetics, and systems modeling, we show that a spatially and temporally changing gradient of Shh signaling is interpreted by the regulatory logic of a downstream transcriptional network. The design of the network, which links three transcription factors to Shh signaling, is responsible for differential spatial and temporal gene expression. In addition, the network renders cells insensitive to fluctuations in signaling and confers hysteresis—memory of the signal. Our findings reveal that morphogen interpretation is an emergent property of the architecture of a transcriptional network that provides robustness and reliability to tissue patterning.

INTRODUCTION

How cell diversity and pattern are generated during tissue development is a long-standing question. Graded signals, often referred to as morphogens, have been suggested to provide the positional information that organizes gene expression and cellular differentiation in many tissues (Grimm et al., 2010; Ibañes and Izpisúa Belmonte, 2008; Lander, 2007). The textbook explanation for their activity is that an extracellular concentration gradient of the morphogen establishes distinct levels of signaling in responding cells and thereby regulates target genes in a concentration-dependent manner (Wolpert et al., 1998). In this view, the pattern of cellular differentiation is a direct and causal readout of a concentration gradient. However, recent studies challenge this idea. First, it is unclear whether a gradient can be sufficiently reliable and precise to pattern a tissue (Bollenbach et al., 2008; Gregor et al., 2007; Kerszberg and Wolpert, 2007; Manu et al., 2009a). Second, evidence from several systems indicates that tissue patterning can take place in the absence of a stable gradient of a morphogen (Harfe et al., 2004; Nahmad and Stathopoulos, 2009; Ochoa-Espinosa et al., 2009). Finally, in addition to the levels of signal, the duration of signaling can contribute to patterning (Ahn and Joyner, 2004; Dessaud et al., 2007; Harfe et al., 2004; Pagès and Kerridge, 2000).

One tissue where these issues are particularly relevant is the vertebrate central nervous system. Here, Sonic Hedgehog (Shh) protein, emanating from the ventrally located notochord and floor plate, forms a gradient (Chamberlain et al., 2008) that is responsible for subdividing the ventral neuroepithelium into five neural progenitor domains, each of which generates distinct neuronal subtypes (Jessell, 2000) (Figure 1A). In vitro, increasing concentrations of Shh ligand or increasing levels of Gli activity, the intracellular transcriptional effectors of Shh signaling, induce successively more ventral neural fates (Dessaud et al., 2007; Ericson et al., 1997; Stamataki et al., 2005). In addition, however, neuronal subtype identity depends on the duration of Shh signaling. Accordingly, more ventral neural progenitor identities require longer durations of Shh signaling (Dessaud et al., 2007; 2010). In vitro studies suggest that cells respond to ongoing exposure to Shh through an adaptation mechanism in which cells become desensitized to Shh (Dessaud et al., 2007). An important question arising from these studies is how progenitors transform dynamic changes in Shh signaling into spatial patterns of gene expression.

The transcriptional network acting downstream of Shh signaling might offer an answer to this question. Roles in the refinement and elaboration of patterning have been identified for the transcriptional circuits engaged in other tissues patterned by morphogens (Davidson, 2010; Davidson and Levine, 2008; Jaeger and Reinitz, 2006; Manu et al., 2009a; Xu et al., 2005). Within the neural tube, three transcription factors, Pax6, Olig2, and Nkx2.2, which identify three spatially distinct ventral progenitor domains, are controlled by Shh signaling (Briscoe et al., 1999; Ericson et al., 1997; Novitch et al., 2001) (Figure 1A). Importantly, the final position of the boundaries of the p3 and pMN progenitor domains is regulated, at least in part, by cross repression between these factors (Briscoe et al., 1999, 2000; Ericson et al., 1997; Novitch et al., 2001). Moreover, these transcription factors

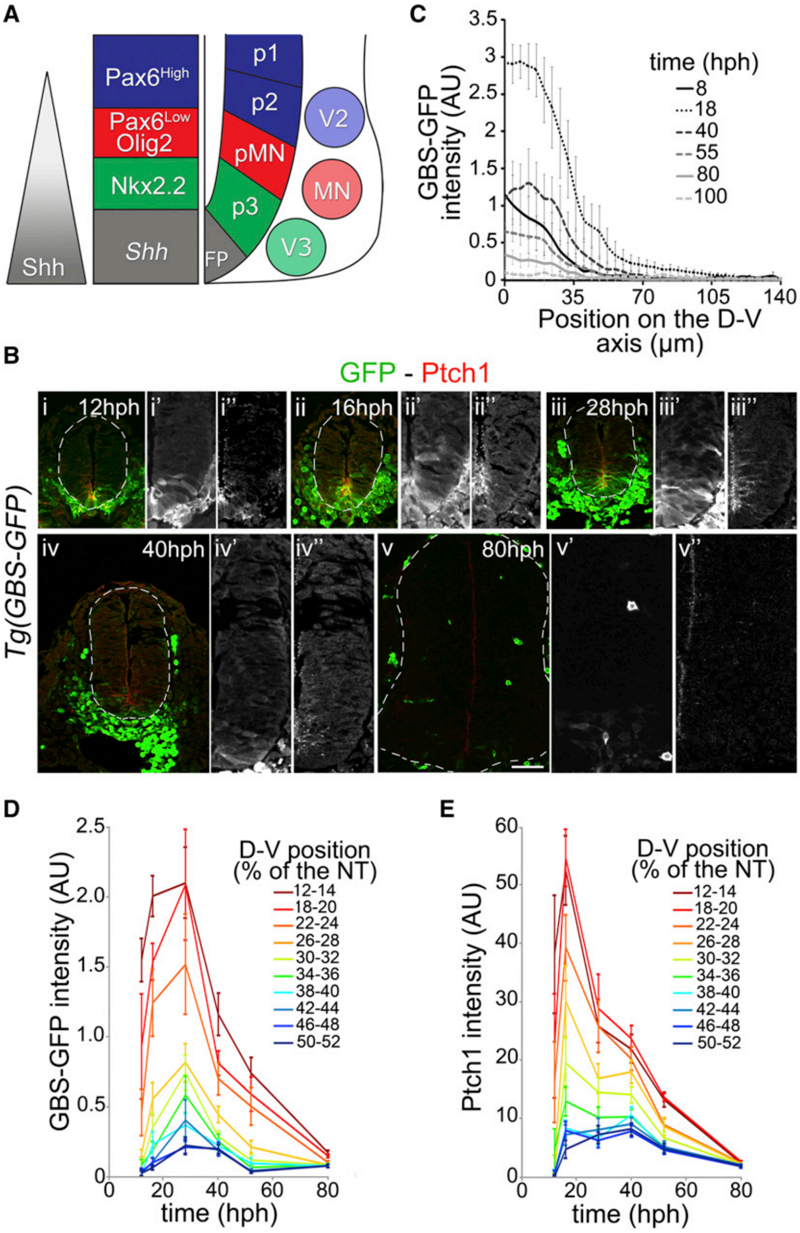

Figure 1. Comparison of Spatial and Temporal Dynamics of Intracellular Shh Signaling and Ptch1 Protein

(A) Shh, secreted from the notochord and floor plate (FP), forms a gradient in the neural tube that divides ventral neural progenitors into molecularly distinct domains. V3 interneurons are generated from Nkx2.2+ p3 progenitors; motor neurons (MN) from Olig2+ pMN progenitors; and V2 neurons are derived from p2 progenitors, expressing Pax6, but not Olig2.

(B) Expression of GFP (green) and Ptch1 (red) at brachial level in *Tg(GBS-GFP)* embryos at the indicated stages.

(C) *Tg(GBS-GFP)* activity (GFP fluorescent intensity in arbitrary unit [AU]; mean ± SD) as a function of dorsal-ventral (D-V) position (μm) in embryos of the indicated stages.

(D) Average *Tg(GBS-GFP)* activity in the neural tube (mean + SEM in arbitrary unit [AU]) at relative distances (percentage [%] of the neural tube) from the floor plate in embryos of the indicated stages (n ≥ 3 embryos/stage).

(E) Quantification of Ptch1 protein (mean fluorescent intensity ± SEM in arbitrary unit [AU]) at relative distances (percentage [%] of the neural tube) from the floor plate in embryos of the indicated stages (n ≥ 3 embryos/stage).

For embryo stages see Table S1. See also Figures S1–S3.

have been suggested to modulate the level of Shh signaling in responding cells as the neural tube is patterned (Lek et al., 2010). Together, these studies identify an important role for the transcriptional circuit in refining the pattern of gene expression in the neural tube, but it leaves unresolved the question of how different levels and durations of Shh signaling control appropriate gene expression in responding cells. Furthermore, the in vivo temporal-spatial profile of Shh signaling and how this produces stable gene expression patterns are unclear.

Here, we use an in vivo reporter of Gli activity to determine the dynamics of Shh signaling in the neural tube, and we provide in silico and in vivo evidence that the regulatory logic of Pax6, Olig2, and Nkx2.2 transcriptional circuit is responsible for interpretation of the Shh signaling gradient. Strikingly, the design of the transcriptional circuit explains both the temporal and graded response to Shh signaling. In addition, it appears to render cells insensitive to transient increases in Shh signaling and produces hysteresis, providing cells with a memory of the signal. Together, these data indicate that the morphogen response of neural cells to Shh is an emergent property of a transcriptional circuit and suggest general principles that are likely to be relevant for morphogen interpretation in many developing tissues.

RESULTS

Dynamics of Intracellular Shh Signaling in Ventral Neural Progenitors

In order to investigate how neural progenitors respond to the Shh gradient (Chamberlain et al., 2008), we first determined the dynamics of downstream intracellular Shh signaling in vivo. To accomplish this, we took advantage of two independent assays of Shh signaling: immunostaining for Ptch1, which is induced by Shh signaling (Goodrich et al., 1997; Marigo et al., 1996; Vokes et al., 2008), and a new transgenic reporter mouse—*Tg(GBS-GFP)*—in which eight concatemerized binding sites for Gli transcription factors regulate GFP expression (Figures S1 and S2 available online).

The profiles of both *Tg(GBS-GFP)* and Ptch1 protein displayed a ventral-to-dorsal gradient (Figures 1, S1D, S1E, S3A, and S3B). *Tg(GBS-GFP)* reporter activity and Ptch1 expression were first detected within the ventral neural tube at ~8 hr postheadfold stage (hph) (Figures 1Bi–1Bi″, 2Ai, and S3Ai–S3Bi). Then, consistent with the progressive increase in the amplitude of the Shh

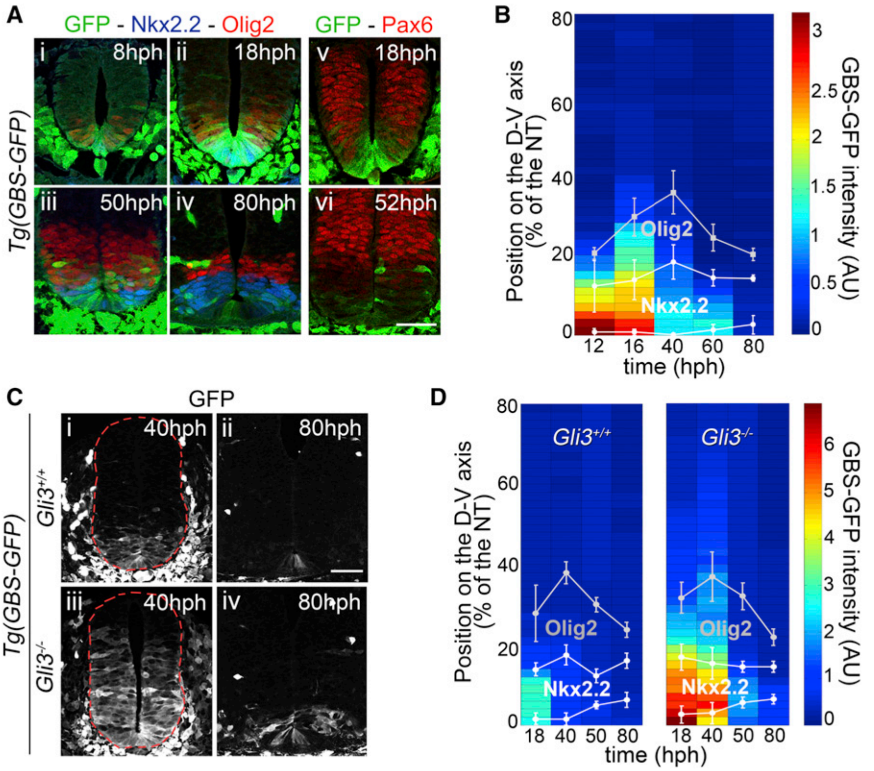

Figure 2. Correlation of Gli Activity and Gene Expression Patterns in Wild-Type and *Gli3* Mutant Embryos

(A) GFP (green), Olig2 (red), Nkx2.2 (blue), and Pax6 (red) at brachial level in *Tg(GBS-GFP)* embryos at the indicated stages.

(B) Heat map of GFP intensity in *Tg(GBS-GFP)* embryos at relative positions measured from the basal side of floor plate cells (percentage [%] of the neural tube) at the indicated stages. The position of the dorsal boundary of Olig2$^+$ and the dorsal and ventral boundaries of Nkx2.2$^+$ domains (mean ± SD) are indicated.

(C) *Tg(GBS-GFP)* activity in brachial regions of the neural tube of 40 and 80 hph *Gli3*$^{+/+}$ and *Gli3*$^{-/-}$ mouse embryos. The red dashed lines outline the pial surface of the neural tube.

(D) Relationship between GFP intensity (AU) in embryos containing *Tg(GBS-GFP)* and dorsal limits of Olig2 and dorsal and ventral boundaries of Nkx2.2$^+$ domain along the DV axis (percentage [%] of the neural tube) in *Gli3*$^{+/+}$ and *Gli3*$^{-/-}$ embryos at the indicated stages.

Scale bars, 50 μm. For embryo stages see Table S1. See also Figure S4.

protein gradient (Chamberlain et al., 2008), the amplitude and range of the gradient of *Tg(GBS-GFP)* activity and Ptch1 expression increased (Figures 1C–1E). The amplitude of the Ptch1 gradient reached a peak between 16 and 20 hph, whereas the *Tg(GBS-GFP)* activity peaked slightly later ~25–30 hph (Figures 1Bii–1Biii″, 1C–1E, 2Aii, S3Aii, and S3Bii). The later timing of peak *Tg(GBS-GFP)* activity could be explained by the relatively long half-life of GFP, estimated to be between 13 and 19 hr (Corish and Tyler-Smith, 1999; data not shown), or differences in the trafficking or transcriptional regulation of Ptch1 and GFP levels.

Following the peak, the amplitude of both *Tg(GBS-GFP)* activity and Ptch1 progressively declined (Figures 1Biii–1Bv″ and 1C–1E). After 70 hph, the expression of GFP protein was barely detectable (Figures 1Bv–1Bv″ and 1D) and from 100 hph was no longer observed (data not shown). Accordingly, the less stable *GFP* mRNA expression confirmed that reporter activity was extinguished by ~55 hph (Figures S3Aiii–S3Av and S4Ciii). Similarly, the amplitude of the Ptch1 protein gradient progressively decreased and by 70 hph reached basal levels 50 times lower than the peak value (Figures 1Biii–1Bv″, 1E, S3Biv, and S3Bv). By 100 hph, Ptch1 protein and mRNA were barely detectable within the most ventral cell types (Figure S3Bv; data not shown). However, in contrast to *Tg(GBS-GFP)* activity, a low level of Ptch1 protein and mRNA was observed in all progenitor cells located dorsal to the p3 domain (Figures 1Bv–1Bv″ and S3Bv). This suggested that either the levels of Gli activity were not detectable by *Tg(GBS-GFP)*, or the maintenance of Ptch1 expression does not require positive Gli activity.

Together, these data indicate that the dynamics of Shh signaling follow an adaptation profile, increasing during early developmental times to reach a peak in e8.5–e9 embryos, then decreasing such that by e10.5 the levels of signaling in the neural tube are low (Figures S1D and S1E). Strikingly, these dynamics of intracellular Shh signaling differ from the gradient of Shh protein, which increases in amplitude over the same time period (Chamberlain et al., 2008), supporting the idea that ventral neural progenitors adapt their response to ongoing Shh exposure (Dessaud et al., 2007; 2010).

Positional Identity Does Not Correspond to Thresholds of Intracellular Signaling

The Shh signaling dynamics prompted us to compare the *Tg(GBS-GFP)* reporter activity to the expression patterns of the downstream genes Pax6, Olig2, and Nkx2.2, the expression of which changes over time (Jeong and McMahon, 2005; Stamataki et al., 2005). At each stage, Nkx2.2 was expressed in regions containing the highest levels of Shh signaling (Figures 2Ai–2Aiv and 2B). At 18 hph, Olig2 and low levels of Pax6 were expressed in cells that contained low levels of Shh signaling (Figures 2Aii, 2Av, and 2B; data not shown). By 50 hph the level of signaling in cells expressing Olig2 and Pax6 had dropped substantially (Figures 2Aiii and 2Avi). High levels of Pax6 were restricted to cells lacking *Tg(GBS-GFP)* activity (Figure 2Avi). These data are consistent with the induction of Nkx2.2 by high and Olig2 by moderate levels of Shh signaling and repression of Pax6 by Shh signaling (Dessaud et al., 2007; Ericson et al., 1997). Crucially, however, over the course of development, the relationship between the level of reporter activity and the expression of each gene changed. For example, the level of GBS-GFP in Olig2-expressing cells was higher at 18 hph than the level of GBS-GFP

activity associated with Nkx2.2 expression at 60 hph (Figure 2B). Moreover, cells at the p3/pMN boundary that received similar levels of Gli activity expressed either Olig2 or Nkx2.2 (Figure 2B). Thus, the induction of Nkx2.2 and Olig2 does not appear to be determined simply by a fixed threshold of Gli activity.

The lack of correlation between thresholds of Gli activity and positional identity was further emphasized by the analysis of embryos lacking Gli3. This Gli protein provides the major transcriptional repressor function in the Shh pathway (Figure S4B; Hui and Joyner, 1993). Consistent with the repressor function of Gli3, the amplitude and range of the *Tg(GBS-GFP)* activity were markedly increased in the neural tube of 40 hph *Gli3* mutant mice (Figures 2Ci, 2Ciii, and 2D). Despite this increase in Gli activity, there was no significant difference in the expression profile of Nkx2.2 in either 40 hph embryos or later embryonic ages (Figures 2D and S4A). Thus, although many cells in the ventral neural tube received levels of Shh signaling that greatly exceeded those usually associated with Nkx2.2 induction, the expression of Nkx2.2 remained confined to its normal spatial domain.

Together, these data demonstrate that the intracellular signaling in response to Shh exposure is highly dynamic and that thresholds of Gli activity are not sufficient to control gene expression. This raises several questions. How do cells acquire their positional identity in response to changing levels of morphogen signaling? What determines the differential response of genes in the neural tube? How are gene expression patterns maintained after the level of signaling has decreased in the neural tube?

The Gene Regulatory Network Produces the Morphogen Response

Pax6, Olig2, and Nkx2.2 are linked together in a gene regulatory network (GRN) that affects their response to Shh signaling (Briscoe et al., 2000; Lek et al., 2010; Novitch et al., 2001; Vokes et al., 2007). A series of regulatory interactions between Pax6, Olig2 and Nkx2.2 have previously been demonstrated to influence the expression of each factor (Briscoe et al., 1999, 2000; Ericson et al., 1997; Novitch et al., 2001). Accordingly, Pax6 represses Nkx2.2, whereas Olig2 inhibits Pax6 expression. Conversely, Nkx2.2 represses Pax6 and Olig2. In addition, a consistent, albeit small, dorsal expansion of Nkx2.2 expression was observed in *Olig2*$^{-/-}$ embryos compared to wild-type stage-matched littermates (Figures 3Aii, 3Avi, 3B, and 4Bii). Conversely, the overexpression of Olig2 in chick neural tube inhibited the induction of Nkx2.2 expression (Figures S5A and S5B). Thus, Olig2 exerts a repressive influence on Nkx2.2 that leads to a revision in the GRN that links these three transcription factors (Figure 4A).

Because GRNs have been shown to control the dynamics and behavior of genes in developing tissues (Davidson, 2010; Davidson and Levine, 2008), we asked whether the regulatory logic linking Shh to Pax6, Olig2, and Nkx2.2 might explain the morphogen response in neural progenitors. Strikingly, the dorsal limit of Nkx2.2 expression at 60 and 80 hph in *Pax6;Olig2* mutant mice expanded to match the dorsal limit of Olig2 expression in equivalent staged wild-type embryos (Figures 3Av, 3Aviii, 3C, 4Bi, and 4Biv). To ask whether this could result from changes in the level of Gli activity in *Pax6;Olig2* mutant mice, we first assayed *Tg(GBS-GFP)* activity in this genetic background. Although the higher levels of *Tg(GBS-GFP)* activity appeared to persist for a somewhat longer time in the most ventral cells of the neural tube in *Pax6;Olig2* mutants, the range of *Tg(GBS-GFP)* activity was unchanged (Figures 3D and 3E; data not shown). More importantly, the level of GFP expression in cells that normally express Olig2 was unchanged from wild-type in *Pax6;Olig2* mutants (Figure 3E; data not shown). This excludes the possibility that Nkx2.2 expansion in the mutants is a consequence of increased Shh signaling. Moreover, RNAi-mediated blockade of Pax6 in neural tube cells resulted in Nkx2.2 induction by levels of Gli activity that were only sufficient to induce Olig2 in wild-type embryos (Figures S5C and S5C′). These results together with the lack of dorsal shift of Nkx2.2 expression domain in *Gli3*$^{-/-}$ mice, despite the increase in Gli activity (Figure 3E), indicate that the differential responses of Nkx2.2 and Olig2 to graded Shh signaling are determined by the regulatory architecture of the transcriptional network, and not by differences in the intrinsic responsiveness of the two genes to Shh signaling.

The GRN Links the Temporal and Graded Responses of Progenitors

Feedback and nonlinearity in even relatively simple gene networks can make their operation difficult to understand (Alon, 2007). Therefore, we formulated a mathematical model of the Pax6-Olig2-Nkx2.2 transcriptional network. Linked ordinary differential equations (ODEs) were used to describe the response of Pax6 (P), Olig2 (O), and Nkx2.2 (N) in time (t) to an input from Shh-Gli signaling (G) (Figure 4A).

$$\frac{dP}{dt}=\frac{\alpha}{1+\left(\frac{N}{N_{critP}}\right)^{h1}+\left(\frac{O}{O_{critP}}\right)^{h2}}-k_1P. \quad (1)$$

$$\frac{dO}{dt}=\frac{\beta G}{1+G}\times\frac{1}{1+\left(\frac{N}{N_{critO}}\right)^{h3}}-k_2O. \quad (2)$$

$$\frac{dN}{dt}=\frac{\gamma G}{1+G}\times\frac{1}{1+\left(\frac{O}{O_{critN}}\right)^{h4}+\left(\frac{P}{P_{critN}}\right)^{h5}}-k_3N. \quad (3)$$

Although this abstraction cannot account for the full complexity of the in vivo situation, it accurately describes the experimentally determined regulatory relationships and allows the logic of these interactions to be explored. Parameter ranges were identified that produced a switch from $P^{HIGH}\rightarrow O^{HIGH}\rightarrow N^{HIGH}$ in response to progressively higher values of G (where X^{HIGH} defines the state in which the value of the indicated variable was above the arbitrary threshold of 1; simulation in Figure 4Bi, see also Experimental Procedures and Table S2). This analysis indicated that the circuit is able to encode the multistate switch, $P^{HIGH}\rightarrow O^{HIGH}\rightarrow N^{HIGH}$, in response to a morphogen-like input. A sensitivity analysis demonstrated that for the majority of parameters, increasing or decreasing their

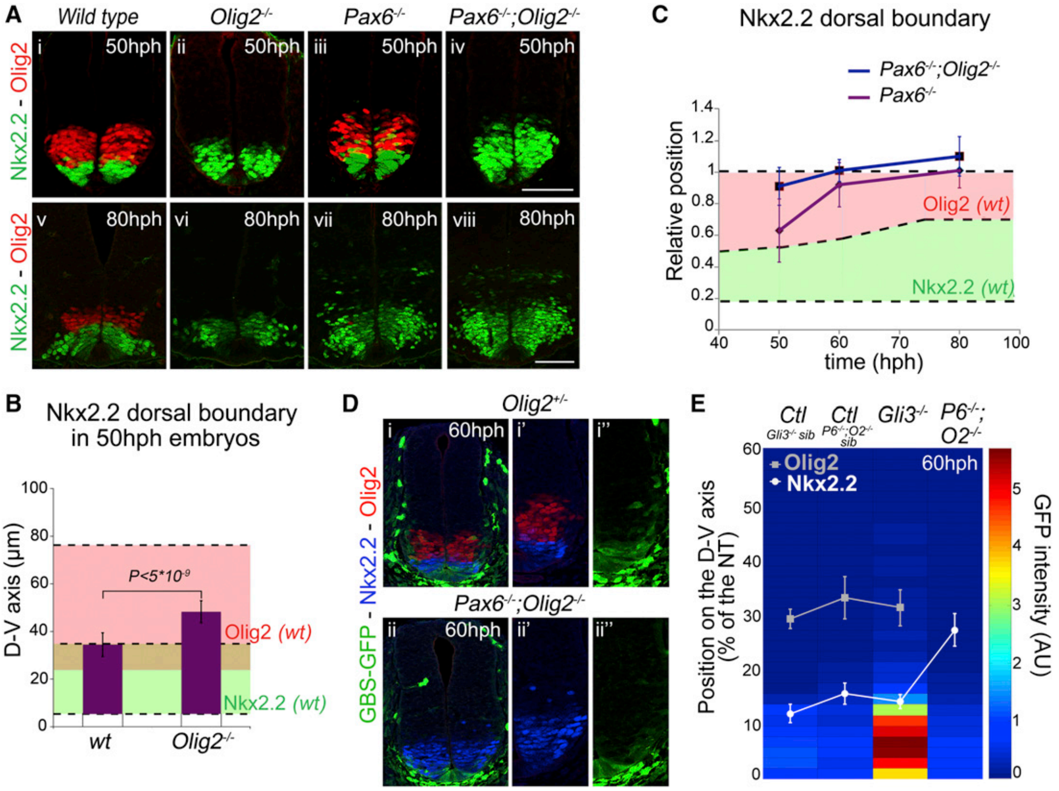

Figure 3. Olig2 and Pax6 Control the Morphogen Response of Nkx2.2 to Shh Signaling

(A) Nkx2.2 (green) and Olig2 (red) expression at forelimb levels of 50 and 80 hph wild-type, *Olig2*$^{-/-}$, *Pax6*$^{-/-}$, and *Pax6*$^{-/-}$*;Olig2*$^{-/-}$ embryos.

(B) Measurements of the dorsal boundary of Nkx2.2 expression in wild-type and *Olig2*$^{-/-}$ embryos at 50 hph (mean ± SD; p values from Student's t test). The average positions of Nkx2.2 (light green) and Olig2 (light red) expression domains in wild-type embryos are indicated.

(C) Measurements of the dorsal boundary position of Nkx2.2 expression in *Pax6*$^{-/-}$ and *Pax6*$^{-/-}$*;Olig2*$^{-/-}$ embryos ($n \geq 3$ embryos; mean ± SD of the Nkx2.2 boundary) at the indicated stages. The normal positions of the Nkx2.2 and Olig2 domains in wild-type embryos are indicated in the shaded regions, and all positions are normalized to that of wild-type Olig2. The Nkx2.2 boundary in *Pax6*$^{-/-}$*;Olig2*$^{-/-}$ mutant embryos is significantly different from wild-type litter-mates (p values from Student's t test, 50 hph: $p < 0.0005$; 60 hph: $p < 5 \times 10^{-9}$; 80 hph: $p < 0.05$).

(D) Nkx2.2 (blue), Olig2 (red), and GFP (green) in forelimb regions of mouse embryos of the indicated genotype and stage ($n \geq 2$ embryos for each data point).

(E) Quantification of the levels of *Tg(GBS-GFP)* activity in *Gli3*, *Pax6;Olig2* mutants, and control wild-type or heterozygous sibling embryos (Ctl *Sib*). Heat maps depict GFP (mean intensity in arbitrary units [AU]) and the dorsal boundaries of Nkx2.2 (white) and Olig2 (gray) (mean ± SD) along the D-V axis (distances from the floor plate in percentage [%] of the neural tube).

Scale bars, 50 μm. For embryo staging see Table S1. See also Figure S5.

value did not affect the behavior of the system, suggesting that when the degradation rates and the repression parameters for all three TFs were of the same order of magnitude, the behavior of the model was robust (Table S3). Importantly, however, for the system to display the appropriate behavior, N had to be the strongest repressor in the circuit, in order to overcome the repression from P and O and prevail in response to high values of G. Analysis of a Heaviside simplification of the system, which allows analytic solutions for the steady states of the system, confirmed the key parameter relationships for the biologically appropriate outputs (J.P., K.M.P., and J.B., unpublished data).

We tested whether the model recapitulated the behavior of gene expression observed in *Olig2*, *Pax6*, or *Pax6;Olig2* mutant embryos (Figures 3A and 4Bii–4Biv). The removal of P or O from the system reduced the three-species network to a two-species cross-repression network similar to those that have been analyzed previously (Cherry and Adler, 2000; Saka and Smith, 2007) and resulted in N achieving its peak activation at lower values of G (Figures 4Bii and 4Biii). Similarly, the removal of P and O resulted in N induction at even lower values of G (Figure 4Biv). These data are consistent with the dorsal expansion of Nkx2.2 expression in the corresponding mouse mutants (Figures 3A and 4Bii–4Biv). Moreover, the removal of P resulted in a more limited induction of O, consistent with the decreased expression of Olig2 observed in *Pax6* mutants (Figure 4Biii).

The in vivo observations (Figure 3C) indicated that, in the absence of Pax6 and Olig2, the extent of Nkx2.2 expression matched that expected of Olig2. In the model, the regulation of Olig2 and Nkx2.2 is simulated in Equations 2 and 3, respectively: in these equations, β and γ determine the maximal rates of expression of O and N, respectively, in response to G. For the induction of N by G, in the model in which O and P are removed,

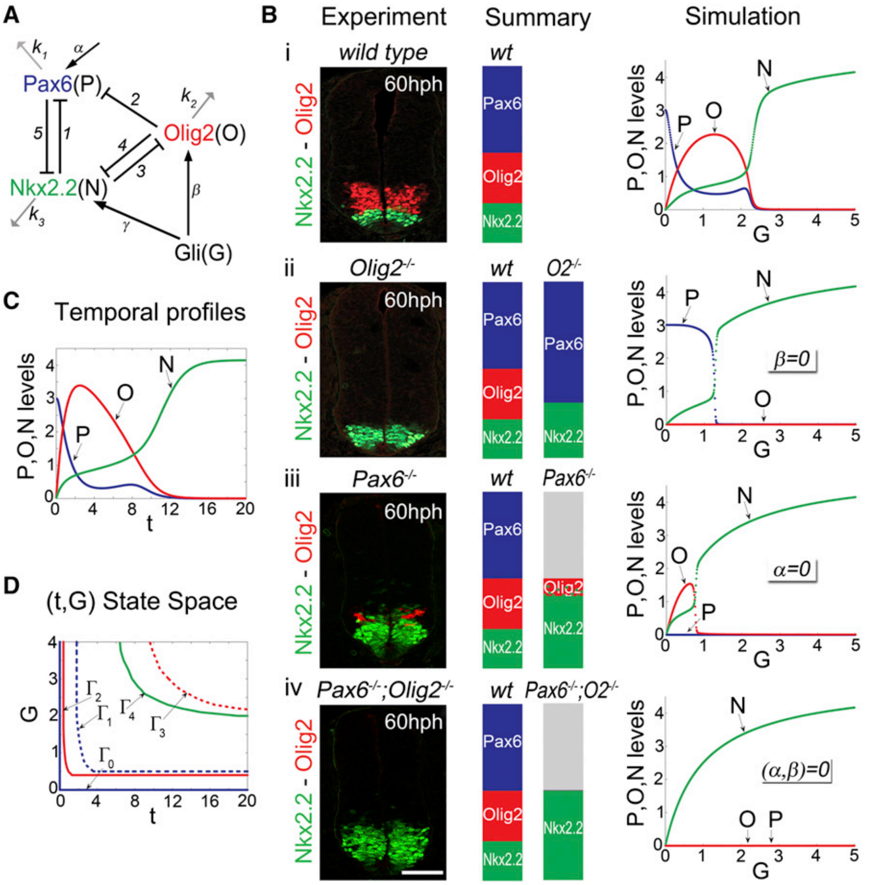

Figure 4. GRN for Shh Morphogen Interpretation

(A) Summary of the genetic network and parameters used for modeling. The regulatory network connects Shh-Gli signaling (G), Pax6 (P), Olig2 (O), and Nkx2.2 (N). "1–5" represents the cross-repressive interactions between the TFs, parameterized by Hill coefficients, h_i, (i = 1–5) and critical values N_{critP}, O_{critP}, N_{critO}, O_{critN}, and P_{critN}. The k_i (i = 1–3) values are degradation rates.

(B) Experiment represents expression of Nkx2.2 (green) and Olig2 (red) in 60 hph brachial neural tube of wild-type and mutant mice. Summary illustrates schematic of expression patterns in the indicated genetic backgrounds. Simulation shows output of the numerical simulations of the model in (A). Values of P, O, and N from numerical simulations plotted as a function of G (for t = 20). Output of the model with P, O, or both P and O removed was obtained with parameter regimes where (α = 0), (β = 0), or (α, β = 0), respectively.

(C) Temporal profile of P, O, and N for G = 5 (t, time).

(D) (t,G) state space of the model indicating the values of G and t for which P, O, or N dominates. Lines (Γ_0–Γ_4) indicate the values of G and t at which P (blue, Γ_0, Γ_1), O (red, Γ_2, Γ_3), N (green, Γ_4) are equal to 1; solid lines indicate the threshold at which the value increases above 1, in the positive t or G direction; dotted lines indicate the threshold at which the value decreases below 1, in the positive t or G direction. The line Γ_0 represents the expression of P when (t, G) = 0.

Scale bars, 50 μm. For embryo stages see Table S1. See also Figure S6, and Tables S2 and S3.

to match the induction of O in the complete model, the value of γ/k_3 must equal β/k_2. Strikingly, simulations of the full model with these parameter conditions indicated that the $P^{HIGH} \rightarrow O^{HIGH} \rightarrow N^{HIGH}$ switch was produced (Tables S2 and S3). Thus, the system, in which the intrinsic response of N and O to G is identical, is sufficient to generate the appropriate tripartite response. These results are counterintuitive because conventional morphogen models predict that the gene requiring higher levels of morphogen signaling should be less sensitive to the signal. Together, therefore, the in silico and in vivo data indicate that the morphogen response of Nkx2.2 and Olig2 to Shh is a property of the regulatory logic of the transcriptional circuit and is unlikely to be established by differential sensitivity of these genes to Shh signaling.

We next examined the temporal behavior of P, O, and N prior to system settling into a stable state. The results showed that for high values of G, a $P^{HIGH} \rightarrow O^{HIGH} \rightarrow N^{HIGH}$ switch, as a function of time, was apparent (Figures 4C and S6A). Hence, there is a correspondence in the dynamic behavior of P, O, N, and the stable states of the system generated by different values of G (Figures 4Bi and 4C). To explore this further, we analyzed the temporal output of the system for different values of G using the same parameter regime. A state space diagram in which the activation of the three species, P, O, N, is a function of time (t) and G (Figures 4D and S6B) indicated that to activate O or N and to repress P, a threshold value of G must be sustained for an appropriate period of time; higher thresholds and longer durations of G are required for induction of N than for O. Moreover, for all levels of signaling, in which the system reached a stable state of N^{HIGH}, an O^{HIGH} state existed transiently prior to N induction (Figure 4D). This behavior is in agreement with empirical observations that Olig2 is expressed in cells prior to Nkx2.2 in vivo and in vitro (Figure 2B; Dessaud et al., 2007; Jeong and McMahon, 2005; Stamataki et al., 2005). In addition, comparison of model simulations in which P, or P and O were removed predicted that the expansion of Nkx2.2 should be more rapid in the absence of both *Pax6* and *Olig2* than in the absence of only *Pax6*—as a consequence of the presence of the repressive activity of Olig2 on Nkx2.2. Consistent with this, the expansion of Nkx2.2 prior to 60 hph was less evident in the *Pax6*$^{-/-}$ embryos than in *Pax6;Olig2* double mutants (Figures 3Aiii, 3Aiv, 3Avii, 3Aviii, and 3C).

Finally, challenging the model with a simulated temporal profile of Gli activity, which mimics the in vivo dynamics of Gli activity, produced the experimentally observed gene expression outputs (Figures S7A and S7C). Together, the analysis indicates that the temporal and graded responses of the system are inseparable, and the stable state to which the system settles is a consequence of the dynamics of regulatory interactions within the network.

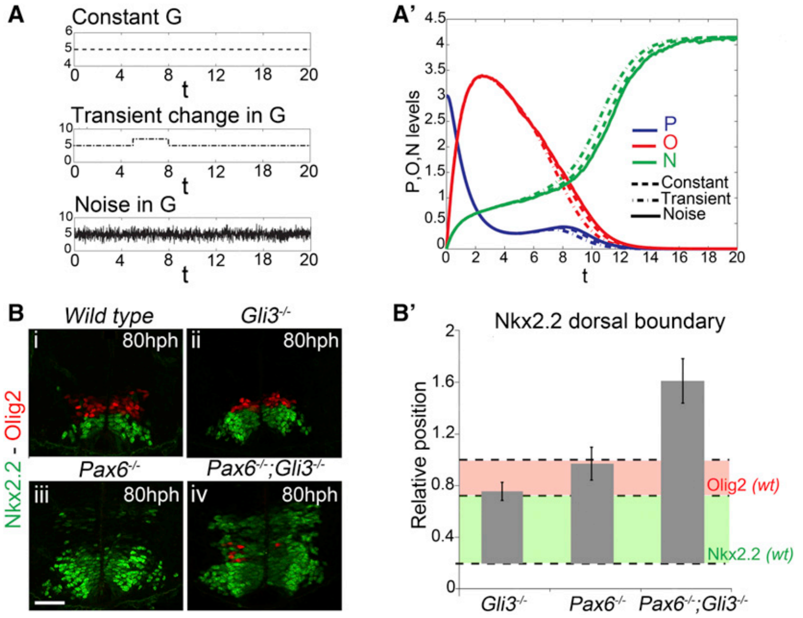

Figure 5. The GRN Buffers Fluctuations in Shh Signaling

(A) Schematic of Gli activity with a constant value, or with a transient increase (step function), or with a white noise term (in all cases, the base level is G = 5; white noise: mean = 5, amplitude = 1).

(A′) Output of the model for a constant G (dashed line), a transient increase in G (dashed-gapped line), and G with a white noise (solid line) (in all cases, the base G = 5).

(B) Nkx2.2 (green) and Olig2 (red) expression at forelimb levels of 80 hph mouse neural tubes from the indicated genotypes.

(B′) Position of the dorsal boundary of the Nkx2.2^{+} domain in *Gli3*$^{-/-}$, *Pax6*$^{-/-}$, and *Pax6*$^{-/-}$;*Gli3*$^{-/-}$ embryos at 80 hph. For comparison the colored shading indicates the Nkx2.2 (light green) and Olig2 (light red) expression in wild-type embryos. All positions are normalized to that of the dorsal limit of wild-type Olig2.

Scale bars, 50 μm. For embryo stages see Table S1.

The Regulatory Logic of the Transcription Circuit Confers Robustness

We next addressed whether the configuration of the network could provide robustness to temporal fluctuations in signal. We first simulated the consequence of a transient increase in Gli activity (using a step function) or the provision of noisy Gli activity (Figure 5A). Introducing these fluctuations in G did not perturb the qualitative output of the system (Figure 5A′). This striking observation suggested that the normal pattern of Nkx2.2 expression in *Gli3* mutants, despite the increase in signaling, might be due to the dynamics of elevated Gli activity in this genetic background. Indeed, examination of Gli activity in *Gli3*$^{-/-}$ embryos, using *Tg(GBS-GFP)*, indicated that the increased signaling was transient, and by 80 hph, GFP distribution in *Gli3* mutants was indistinguishable from control embryos (Figures 2C, 2D, S4A, and S4C). The return of Gli activity to normal levels in *Gli3*$^{-/-}$ can be explained by the mechanism of adaptation of cells to Shh signaling. The higher levels of signaling produced in the absence of *Gli3* resulted in strong upregulation of Ptch1 expression (Figure S4B). This provides additional negative feedback that would act homeorhetically to restore signaling to normal levels. Consistent with this, increasing Smo activity by culturing embryos with Pur for 6 hr transiently induced high levels of *Tg(GBS-GFP)* activity but had no effect on the patterning of the ventral neural tube (Figures S2D and S2E). Together, these data strongly support the idea that sustained levels of Shh signaling are required for Nkx2.2 induction and suggest that the transcriptional circuit acts as a buffer to transient increases in signaling.

To test this idea in vivo, we asked whether perturbation of the circuit sensitized the neural tube to increased Shh signaling. To this end, we assayed embryos lacking both *Pax6* and *Gli3*. In these embryos there was a much greater dorsal expansion in the domain of Nkx2.2 expression compared to the absence of *Pax6* or *Gli3* alone (Figures 5B and 5B′). Thus, increased Shh signaling in the absence of Pax6 markedly increased the range of Nkx2.2 induction. This is consistent with the importance of the regulatory circuit to buffer transient fluctuations in signaling and offers an explanation for the robustness of patterning in the ventral neural tube.

Hysteresis in the Response of Nkx2.2 to Shh Signaling

Model simulations suggested that the circuit should confer hysteresis on Nkx2.2 in response to Shh signaling (Figure 6A). Accordingly, the value of G necessary to maintain N, once activated, was lower than that needed to initially induce N. Examination of the parameters suggested that this would happen in conditions in which N is sufficient to inhibit both P and O, resulting in an effective positive feedback loop (sometimes called double negative) between N and P (Figure 7Bv). Generically, such networks lead to bistability and hysteresis (Tyson and Othmer, 1978). By contrast we identified parameter sets in which oscillatory behavior, rather than hysteresis, could be observed as the system switched from P^{HIGH} to O^{HIGH} to N^{HIGH} (Figures S7B and S7B′). Such periodic behavior was evident in the switch from O^{HIGH} to N^{HIGH} and occurred when the parameter values were such that neither P nor O or N prevailed over all the other TFs (Table S2). In these cases, the network is effectively a negative feedback loop resembling a repressilator (Elowitz and Leibler, 2000). Importantly, the parameter sets that generate hysteresis or repressilator-like oscillations were mutually exclusive; thus, the presence of hysteresis would rule out oscillations (J.P., K.M.P., and J.B., unpublished data).

To test for hysteresis in neural progenitors, we assayed Nkx2.2 in explants of intermediate regions of naive chick neural plate (Dessaud et al., 2007; 2010; Ericson et al., 1997) exposed to recombinant Shh protein (Figures 6B–6D′). Treatment with 4 nM Shh generated high levels of Gli activity and induced Nkx2.2 expression in most cells by 18 hr (Figures 6C, 6Di, 6Dii, 6D′i, and 6D′ii). Low levels of Gli activity produced by exposure to a combination of 4 nM Shh and 50 nM cyclopamine (Cyc), an

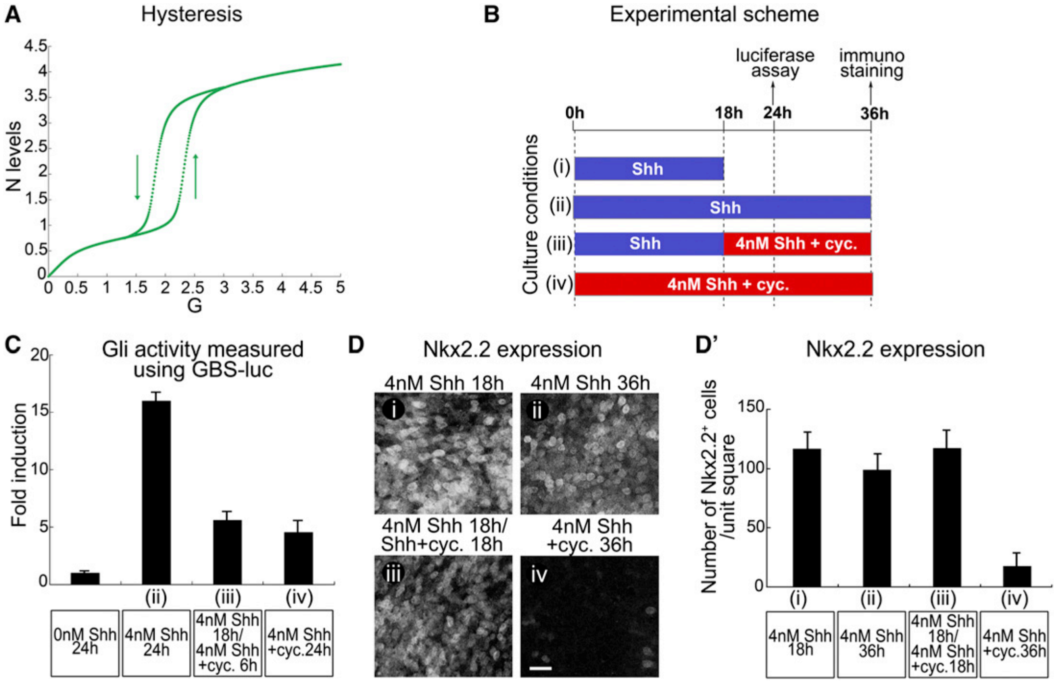

Figure 6. The GRN Confers Hysteresis

(A) A plot of N as a function of G illustrating bistability (t = 20).

(B) Schematic of the experiments in (C) and (D). (i) Explants were incubated with 4 nM Shh for 18 hr, (ii) 4 nM Shh for 36 hr, (iii) 4 nM Shh for 18 hr followed by 4 nM Shh plus 50 nM cyclopamine (Cyc) for 18 hr, or (iv) 4 nM Shh plus 50 nM Cyc for 36 hr.

(C) Gli activity (relative Gli activity ± SEM) measured with GBS-luc in (i) explants treated in the indicated conditions for 24 hr. Gli activity is plotted relative to the activity in explants cultured in the absence of Shh.

(D) Nkx2.2 expression in (i) explants cultured in the indicated conditions (Scale bars, 20 μm).

(D′) Number of cells expressing Nkx2.2 in (i) explants in the indicated conditions (n ≥ 3 explants; two units/explant; number of cells per unit ± SD).

antagonist of Shh signaling (Cooper et al., 1998), did not induce Nkx2.2 and resulted in cells adopting an Olig2 identity (Figures 6Biv–6Div′; data not shown). By contrast, if the levels of Gli activity were reduced, by addition of 50 nM Cyc, after 18 hr of exposure to 4 nM Shh (Figures 6Biii and 6Ciii), Nkx2.2 expression was sustained (compare Figure 6Diii with 6Di). Nevertheless, the maintenance of Nkx2.2 required Gli activity because the complete blockade of signaling with 500 nM Cyc at 18 hr inhibited Nkx2.2 expression (Dessaud et al., 2007; 2010). Thus, lower levels of Shh signaling are required to sustain than to induce Nkx2.2 expression, consistent with the gene regulatory circuit conferring hysteresis. Importantly, these experiments suggest an explanation for the persistence of Nkx2.2 expression in the p3 domain in vivo, despite the level of Gli activity and Gli1 and Gli2 expression in these cells decreasing with developmental age (Figures 2B, S3C, and S3D).

DISCUSSION

We provide evidence that Shh morphogen interpretation in the neural tube is an emergent property of its downstream GRN. Cells transform the extracellular gradient of Shh into a dynamic profile of intracellular Gli activity that engages a transcriptional circuit, the regulatory logic of which is responsible for the generation of the characteristic temporal and spatial patterns of gene expression (Figure 7). This mechanism offers a powerful strategy to achieve the characteristic precision and robustness of morphogen-mediated pattern formation.

Adapting Dynamics of Gli Activity In Vivo

Previous in vitro studies predict that neural cells adapt to continuous exposure to Shh by progressively becoming less responsive (Dessaud et al., 2007; 2010; Jeong and McMahon, 2005). The analysis of Gli activity and Ptch1 expression revealed a similar desensitization in vivo (Figure 1). These dynamics of Gli activity provide a contrast with other morphogens in which signaling appears to remain constant during the patterning phase (Gregor et al., 2007) or increase with time (Bergmann et al., 2007; Harvey and Smith, 2009; Wartlick et al., 2011).

As a negative regulator of the Shh pathway, Ptch1 is likely to contribute to the nonlinear transduction of Shh signaling (Dessaud et al., 2007; Goodrich et al., 1997; Jeong and McMahon, 2005). Consistent with this, the amplitude of the Shh gradient increases in the absence of feedback (Chamberlain et al., 2008), and Ptch1 transcript and protein are strongly upregulated in a spatial and temporal profile that matches the dynamics of Gli activity (Figures 1E and S3B). Patched has also been implicated in shaping the gradient of Hh signaling in the *Drosophila* wing disc (Chen and Struhl, 1996; Nahmad and Stathopoulos, 2009). However, in this tissue the Hh-dependent upregulation

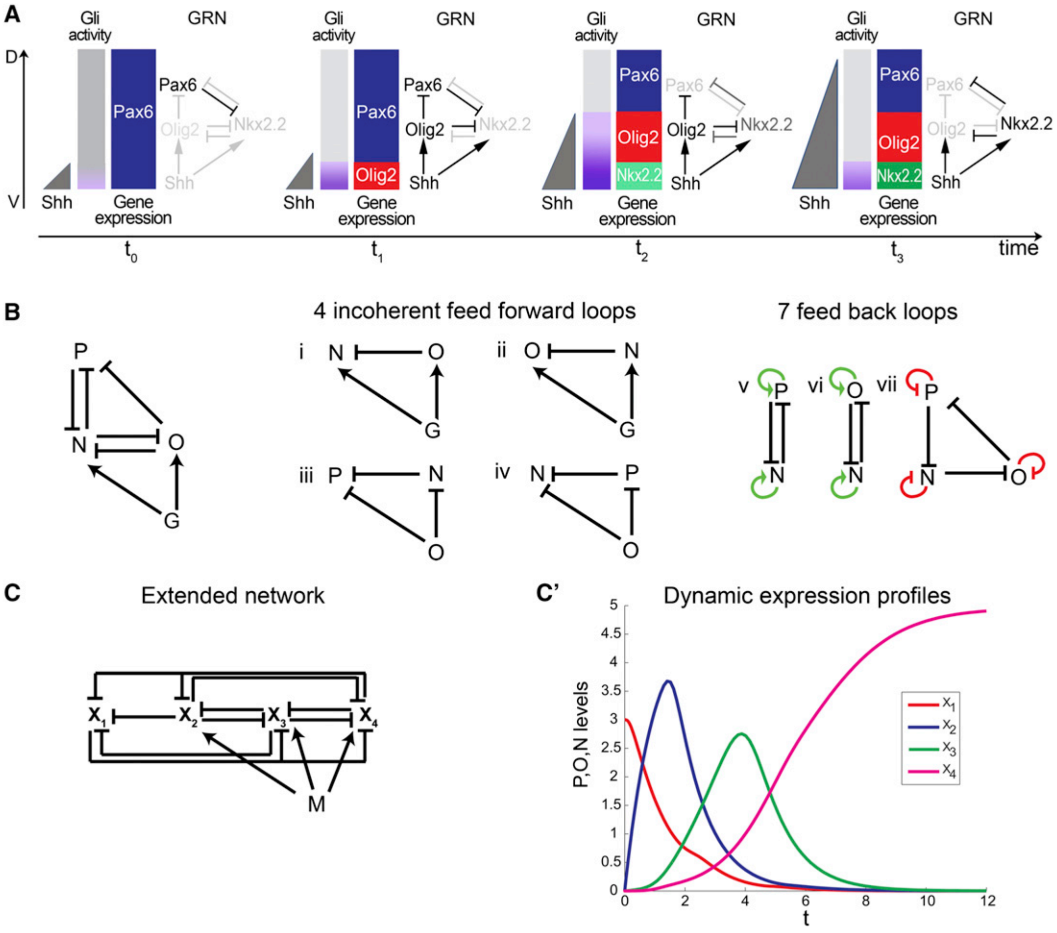

Figure 7. A Model for Morphogen Interpretation

(A) Schematic of Shh signaling-mediated patterning of the ventral neural tube. At t_0, low levels of Shh protein, emanating from the notochord, are translated into low levels of intracellular Gli activity, which are not sufficient to induce Olig2 and Nkx2.2 or to repress Pax6. As development progresses, increasing production of Shh ligand generates a gradient of Gli activity that increases in amplitude (t_1), then reaches a peak (t_2) before retracting (t_3). Gli activity is interpreted by ventral progenitors by the GRN: Olig2 is initially induced (t_1) and represses Pax6. Subsequently, Nkx2.2 is induced (t_2) and represses Pax6 and Olig2. Hysteresis maintains these domains of expression as the amplitude of the Gli activity decreases (t_3).

(B) The regulatory circuit connecting G, N, O, and P is composed of four interconnected incoherent feed forward loops (IFFL). These are type 1 and type 2 IFFLs (Alon, 2007). Two type 1 IFFL link G, N, and O (i and ii), whereas two type 2 IFFL connect P, N, and O (iii and iv). The arrangement results in each factor receiving positive (v and vi, green arrows) and negative feedback (vii, red blunt arrows).

(C) A generalization of the 3-transcription factor gene regulatory circuit and an extension of the network to include an additional component. M is the morphogen signal and X_i the transcription factors. The additional transcription factor is added to the network by two interconnected type 1 IFFL.

(C′) Graph depicting the long-time steady-state profiles of X_1 (red), X_2 (blue), X_3 (green), and X_4 (pink) from the system in (B) and (C).

See also Figure S7.

of Ptch is proposed to bind and sequester Hh, thereby nonautonomously reducing ligand spread (Chen and Struhl, 1996; Nahmad and Stathopoulos, 2009). This mechanism is unlikely to play the major role in the dynamics of Gli activity in the neural tube because the amplitude and range of the Shh gradient increase during the period of time that the Ptch1 and Gli activity gradients decrease (Chamberlain et al., 2008).

Other mechanisms are also likely to contribute to the observed Gli activity dynamics. Embryos in which Shh signaling is activated by an agonist of Smo, which bypasses Ptch1-mediated negative feedback, showed a progressive downregulation of Shh signaling following an initial transient burst (Figure S2E). The inhibition of Gli gene expression in progenitors exposed to Shh (Lek et al., 2010; Matise et al., 1998) could explain, at least in part, a decrease in signaling. Taken together, therefore, the data support the idea that cell autonomous feedback contributes to the temporal adaptation of Gli activity.

Morphogen Interpretation as an Emergent Property of a Transcriptional Network

Our analysis indicates that the downstream transcriptional network is responsible for morphogen interpretation. The correspondence between the wild-type domain of Olig2 expression and the domain of Nkx2.2 expression in *Pax6*$^{-/-}$;*Olig2*$^{-/-}$

embryos indicates that in the absence of repression by Pax6 and Olig2, the response of Nkx2.2 and Olig2 to Shh is similar. Thus, instead of different intrinsic responsiveness of the target genes to morphogen (Driever et al., 1989; Jiang et al., 1991), the regulatory logic of the transcriptional circuit determines the pattern of expression of each gene. In other words, higher levels of Gli activity are required to induce Nkx2.2 than Olig2 because Nkx2.2 repression by Pax6 and Olig2 must be overcome. In silico analysis confirms that the circuit can interpret a morphogen even when the genes are equally responsive to the signal. In this view, the morphogen response emerges from the design of the transcriptional circuit rather than being encoded in discrete parts of the system.

It is tempting to hypothesize that morphogen-controlled GRNs may be the main driver of pattern formation in other tissues. The transcriptional circuit composed of Gap genes that operates along the anterior-posterior axis of the *Drosophila* embryo appears to refine and stabilize the patterns of gene expression generated by differential responses to a gradient of Bicoid (Manu et al., 2009b). Moreover, an analysis of genes responding to Bicoid failed to find a correlation between the affinity and number of binding sites for Bicoid in the regulatory elements of these genes and their pattern of expression along the anterior-posterior axis (Ochoa-Espinosa et al., 2005). Similarly, the level of the Dorsal morphogen does not appear to be directly related to the response of target genes along the dorsal-ventral axis of the embryo (Liberman et al., 2009), and regulatory interactions between the transcription factors controlled by Dorsal have been implicated in refining spatial patterns of gene expression (Stathopoulos and Levine, 2005).

The regulatory logic of the Pax6-Olig2-Nkx2.2 circuit explains why both the level and duration of Shh signaling affect gene expression. Two factors dictate the response of a cell: the current level of Gli activity, and the existing gene expression profile in the cell (Figure 7A). Because the current state of gene expression in a cell is a consequence of prior Gli activity, which results from exposure to Shh, it provides a memory of the signaling experienced by a cell. In this way the circuit acts as the timer that measures the duration, as well as the level, of Gli activity and Shh exposure. The consequence of this mechanism is that the response to different levels of signal is produced by the same mechanism that generates the different temporal responses of the genes. Thus, the temporal and graded responses to Shh are inseparable properties of the regulatory circuit. This reconciles experimental results that have indicated that either the level or the duration of signaling is critical for the control of gene expression (Ahn and Joyner, 2004; Dessaud et al., 2007; 2010; Harfe et al., 2004; Pagès and Kerridge, 2000). Moreover, the observation that temporally changing levels of signaling can generate the same spatial patterns of gene expression suggests that different modes of signaling—temporal versus graded—could be responsible for the profile of gene expression at different positions within the tissue.

The Transcriptional Network Confers Robustness and Memory

In addition to explaining the mechanism that results in differential spatial patterns of gene expression, we provide in silico and in vivo evidence that the network confers both robustness to signal fluctuation and hysteresis. The insensitivity of the circuit to transient changes in the level of signaling provides a means to achieve reliable patterning despite the inherent noisiness of development (Bollenbach et al., 2008; Gregor et al., 2007). This suggests a solution to the apparent discrepancy between the accuracy of patterning processes and the limits on the precision of morphogen gradients (Bollenbach et al., 2008; Gregor et al., 2007; Jaeger and Reinitz, 2006; Manu et al., 2009a). Moreover, this might help explain why, in the absence of Gli3, although there is a marked increase in signaling, only minor neural tube patterning defects are observed. We provide evidence that the transcriptional circuit is able to buffer the short-lived elevation in signaling levels that result from loss of Gli3. The increased levels of Ptch1, induced by the increased signaling, are then likely to contribute to restoring signaling to normal levels. This represents an example of system-level feedback and, from the perspective of Waddington's epigenetic landscape (Waddington, 1942), suggests a molecular explanation for the phenomenon of "canalization," although further studies will be necessary to identify additional mechanisms for the robustness of neural tube patterning.

The in silico and experimental analysis also revealed that the transcriptional circuit confers hysteresis to Nkx2.2 expression. This offers an explanation for how the pattern of gene expression is maintained during development even as the signaling gradient recedes. The maintenance of gene expression patterns during the elaboration of tissue development is a key feature of patterning. The finding that the same mechanism is responsible for both the initial interpretation and the maintenance of Nkx2.2 provides an elegant solution to this problem.

Finally, examination of the regulatory logic of the circuit revealed that it consists of an overlapping arrangement of positive and negative feedback (Figure 7B). Together with the experimental analysis, this provides an intuitive understanding of the performance of the transcriptional circuit. Moreover, the logic can be generalized and extended to regulate additional target genes in a morphogen-like manner (Figures 7C and 7C′). As such, this mechanism might represent a general strategy for morphogen interpretation. Together, the study highlights the information-processing power of transcriptional networks (Alon, 2007; Davidson, 2010; Jaeger and Reinitz, 2006; Vokes et al., 2007), and the simplicity and adaptability of this mechanism suggest that it is likely to be relevant for the control of patterning of tissues other than the neural tube.

EXPERIMENTAL PROCEDURES

Mouse Lines

Mice containing mutant alleles for *Pax6* (*Small Eye* allele), *Olig2*, and *Gli3* (*Xt^J* allele) have been described previously (Ericson et al., 1997; Hui and Joyner, 1993; Zhou and Anderson, 2002). To generate the *Tg(GBS-GFP)* line, eight concatemerized fragments of a FoxA2 enhancer that contains a Gli binding site (Sasaki et al., 1997) were cloned upstream of the *hsp68* minimal promoter and *eGFP* (details are provided in Extended Experimental Procedures). To stage mice, we used somite number and converted these to standardized times (Table S1) expressed as hours postheadfold stage (hph). All procedures were carried out with the approval of the Institute Ethical and Biological Services Animal Research Committees under Home Office Project License (PPL 80/2091).

Immunohistochemistry and In Situ Hybridization

Mouse embryos from timed pregnant females were staged and fixed in 4% paraformaldehyde for 45 min to 2 hr at 4°C. Fixed embryos were cryoprotected by equilibration in 15% sucrose, cryosectioned (14 μm), and processed for immunostaining (Briscoe et al., 2000) or in situ hybridization (ISH) (Yamada et al., 1993). Details of the reagents and the quantification are provided in the Extended Experimental Procedures.

Chick Neural Plate Explant Culture

Neural plate tissue was isolated from HH10 stage chick embryos and cultured as described (Yamada et al., 1993). Shh protein was generated as described (Ericson et al., 1997). Cyc (Toronto Research Chemicals) was dissolved in 100% ethanol. Luciferase assays in explants were performed as previously described (Dessaud et al., 2007). Each experiment was performed independently more than once and gave reproducible results.

SUPPLEMENTAL INFORMATION

Supplemental Information includes Extended Experimental Procedures, seven figures, and three tables and can be found with this article online at doi:10.1016/j.cell.2011.10.047.

ACKNOWLEDGMENTS

We are grateful to N. Bushati, A. Kicheva, T.M. Jessell, E. Kutejova, S. Tozer, and J.P. Vincent for discussions. We thank NIMR Biological Services staff for help with the mouse colonies. This work was supported by the MRC (U117560541) and Wellcome Trust (080630). E.D. was supported by EMBO and Marie Curie Fellowships, N.S. by a Marie Curie Fellowship, V.R. by an EMBO Fellowship, and A.R. by the FCT, Portugal. K.M.P. thanks the National Institute of Mathematical and Biological Synthesis for a sabbatical fellowship (NSF Grant EF-0832858 and University of Tennessee, Knoxville).

N.B. and E.D. conceived and initiated the study. V.R. generated Tg(GBS-GFP) mice. A.R. and V.R designed and performed the quantitative analyses of mouse embryos. N.B and V.R performed mouse genetic experiments. N.S. designed and performed explant experiments. J.P. and K.M.P formulated and analyzed the mathematical model. V.R., N.B., and J.B. wrote the manuscript.

Received: March 10, 2011
Revised: August 14, 2011
Accepted: October 18, 2011
Published: January 19, 2012

REFERENCES

Ahn, S., and Joyner, A.L. (2004). Dynamic changes in the response of cells to positive hedgehog signaling during mouse limb patterning. Cell *118*, 505–516.

Alon, U. (2007). Network motifs: theory and experimental approaches. Nat. Rev. Genet. *8*, 450–461.

Bergmann, S., Sandler, O., Sberro, H., Shnider, S., Schejter, E., Shilo, B.Z., and Barkai, N. (2007). Pre-steady-state decoding of the Bicoid morphogen gradient. PLoS Biol. *5*, e46.

Bollenbach, T., Pantazis, P., Kicheva, A., Bökel, C., González-Gaitán, M., and Jülicher, F. (2008). Precision of the Dpp gradient. Development *135*, 1137–1146.

Briscoe, J., Sussel, L., Serup, P., Hartigan-O'Connor, D., Jessell, T.M., Rubenstein, J.L., and Ericson, J. (1999). Homeobox gene Nkx2.2 and specification of neuronal identity by graded Sonic hedgehog signalling. Nature *398*, 622–627.

Briscoe, J., Pierani, A., Jessell, T.M., and Ericson, J. (2000). A homeodomain protein code specifies progenitor cell identity and neuronal fate in the ventral neural tube. Cell *101*, 435–445.

Chamberlain, C.E., Jeong, J., Guo, C., Allen, B.L., and McMahon, A.P. (2008). Notochord-derived Shh concentrates in close association with the apically positioned basal body in neural target cells and forms a dynamic gradient during neural patterning. Development *135*, 1097–1106.

Chen, Y., and Struhl, G. (1996). Dual roles for patched in sequestering and transducing Hedgehog. Cell *87*, 553–563.

Cherry, J.L., and Adler, F.R. (2000). How to make a biological switch. J. Theor. Biol. *203*, 117–133.

Cooper, M.K., Porter, J.A., Young, K.E., and Beachy, P.A. (1998). Teratogen-mediated inhibition of target tissue response to Shh signaling. Science *280*, 1603–1607.

Corish, P., and Tyler-Smith, C. (1999). Attenuation of green fluorescent protein half-life in mammalian cells. Protein Eng. *12*, 1035–1040.

Davidson, E.H. (2010). Emerging properties of animal gene regulatory networks. Nature *468*, 911–920.

Davidson, E.H., and Levine, M.S. (2008). Properties of developmental gene regulatory networks. Proc. Natl. Acad. Sci. USA *105*, 20063–20066.

Dessaud, E., Yang, L.L., Hill, K., Cox, B., Ulloa, F., Ribeiro, A., Mynett, A., Novitch, B.G., and Briscoe, J. (2007). Interpretation of the sonic hedgehog morphogen gradient by a temporal adaptation mechanism. Nature *450*, 717–720.

Dessaud, E., Ribes, V., Balaskas, N., Yang, L.L., Pierani, A., Kicheva, A., Novitch, B.G., Briscoe, J., and Sasai, N. (2010). Dynamic assignment and maintenance of positional identity in the ventral neural tube by the morphogen sonic hedgehog. PLoS Biol. *8*, e1000382.

Driever, W., Thoma, G., and Nüsslein-Volhard, C. (1989). Determination of spatial domains of zygotic gene expression in the *Drosophila* embryo by the affinity of binding sites for the bicoid morphogen. Nature *340*, 363–367.

Elowitz, M.B., and Leibler, S. (2000). A synthetic oscillatory network of transcriptional regulators. Nature *403*, 335–338.

Ericson, J., Rashbass, P., Schedl, A., Brenner-Morton, S., Kawakami, A., van Heyningen, V., Jessell, T.M., and Briscoe, J. (1997). Pax6 controls progenitor cell identity and neuronal fate in response to graded Shh signaling. Cell *90*, 169–180.

Goodrich, L.V., Milenković, L., Higgins, K.M., and Scott, M.P. (1997). Altered neural cell fates and medulloblastoma in mouse patched mutants. Science *277*, 1109–1113.

Gregor, T., Tank, D.W., Wieschaus, E.F., and Bialek, W. (2007). Probing the limits to positional information. Cell *130*, 153–164.

Grimm, O., Coppey, M., and Wieschaus, E. (2010). Modelling the Bicoid gradient. Development *137*, 2253–2264.

Harfe, B.D., Scherz, P.J., Nissim, S., Tian, H., McMahon, A.P., and Tabin, C.J. (2004). Evidence for an expansion-based temporal Shh gradient in specifying vertebrate digit identities. Cell *118*, 517–528.

Harvey, S.A., and Smith, J.C. (2009). Visualisation and quantification of morphogen gradient formation in the zebrafish. PLoS Biol. *7*, e1000101.

Hui, C.C., and Joyner, A.L. (1993). A mouse model of greig cephalopolysyndactyly syndrome: the extra-toesJ mutation contains an intragenic deletion of the Gli3 gene. Nat. Genet. *3*, 241–246.

Ibañes, M., and Izpisúa Belmonte, J.C. (2008). Theoretical and experimental approaches to understand morphogen gradients. Mol. Syst. Biol. *4*, 176.

Jaeger, J., and Reinitz, J. (2006). On the dynamic nature of positional information. Bioessays *28*, 1102–1111.

Jeong, J., and McMahon, A.P. (2005). Growth and pattern of the mammalian neural tube are governed by partially overlapping feedback activities of the hedgehog antagonists patched 1 and Hhip1. Development *132*, 143–154.

Jessell, T.M. (2000). Neuronal specification in the spinal cord: inductive signals and transcriptional codes. Nat. Rev. Genet. *1*, 20–29.

Jiang, J., Kosman, D., Ip, Y.T., and Levine, M. (1991). The dorsal morphogen gradient regulates the mesoderm determinant twist in early *Drosophila* embryos. Genes Dev. *5*, 1881–1891.

Kerszberg, M., and Wolpert, L. (2007). Specifying positional information in the embryo: looking beyond morphogens. Cell *130*, 205–209.

Lander, A.D. (2007). Morpheus unbound: reimagining the morphogen gradient. Cell *128*, 245–256.

Lek, M., Dias, J.M., Marklund, U., Uhde, C.W., Kurdija, S., Lei, Q., Sussel, L., Rubenstein, J.L., Matise, M.P., Arnold, H.H., et al. (2010). A homeodomain feedback circuit underlies step-function interpretation of a Shh morphogen gradient during ventral neural patterning. Development *137*, 4051–4060.

Liberman, L.M., Reeves, G.T., and Stathopoulos, A. (2009). Quantitative imaging of the Dorsal nuclear gradient reveals limitations to threshold-dependent patterning in *Drosophila*. Proc. Natl. Acad. Sci. USA *106*, 22317–22322.

Manu, S., Surkova, S., Spirov, A.V., Gursky, V.V., Janssens, H., Kim, A.R., Radulescu, O., Vanario-Alonso, C.E., Sharp, D.H., Samsonova, M., and Reinitz, J. (2009a). Canalization of gene expression and domain shifts in the *Drosophila* blastoderm by dynamical attractors. PLoS Comput. Biol. *5*, e1000303.

Manu, S., Surkova, S., Spirov, A.V., Gursky, V.V., Janssens, H., Kim, A.R., Radulescu, O., Vanario-Alonso, C.E., Sharp, D.H., Samsonova, M., and Reinitz, J. (2009b). Canalization of gene expression in the *Drosophila* blastoderm by gap gene cross regulation. PLoS Biol. *7*, e1000049.

Marigo, V., Scott, M.P., Johnson, R.L., Goodrich, L.V., and Tabin, C.J. (1996). Conservation in hedgehog signaling: induction of a chicken patched homolog by Sonic hedgehog in the developing limb. Development *122*, 1225–1233.

Matise, M.P., Epstein, D.J., Park, H.L., Platt, K.A., and Joyner, A.L. (1998). Gli2 is required for induction of floor plate and adjacent cells, but not most ventral neurons in the mouse central nervous system. Development *125*, 2759–2770.

Nahmad, M., and Stathopoulos, A. (2009). Dynamic interpretation of hedgehog signaling in the *Drosophila* wing disc. PLoS Biol. *7*, e1000202.

Novitch, B.G., Chen, A.I., and Jessell, T.M. (2001). Coordinate regulation of motor neuron subtype identity and pan-neuronal properties by the bHLH repressor Olig2. Neuron *31*, 773–789.

Ochoa-Espinosa, A., Yucel, G., Kaplan, L., Pare, A., Pura, N., Oberstein, A., Papatsenko, D., and Small, S. (2005). The role of binding site cluster strength in Bicoid-dependent patterning in *Drosophila*. Proc. Natl. Acad. Sci. USA *102*, 4960–4965.

Ochoa-Espinosa, A., Yu, D., Tsirigos, A., Struffi, P., and Small, S. (2009). Anterior-posterior positional information in the absence of a strong Bicoid gradient. Proc. Natl. Acad. Sci. USA *106*, 3823–3828.

Pagès, F., and Kerridge, S. (2000). Morphogen gradients. A question of time or concentration? Trends Genet. *16*, 40–44.

Saka, Y., and Smith, J.C. (2007). A mechanism for the sharp transition of morphogen gradient interpretation in Xenopus. BMC Dev. Biol. *7*, 47.

Sasaki, H., Hui, C., Nakafuku, M., and Kondoh, H. (1997). A binding site for Gli proteins is essential for HNF-3beta floor plate enhancer activity in transgenics and can respond to Shh in vitro. Development *124*, 1313–1322.

Stamataki, D., Ulloa, F., Tsoni, S.V., Mynett, A., and Briscoe, J. (2005). A gradient of Gli activity mediates graded Sonic Hedgehog signaling in the neural tube. Genes Dev. *19*, 626–641.

Stathopoulos, A., and Levine, M. (2005). Genomic regulatory networks and animal development. Dev. Cell *9*, 449–462.

Tyson, J.J., and Othmer, H.G. (1978). The dynamics of feedback control circuits in biochemical pathways. In Progress in Theoretical Biology, *Volume 5*, R. Rosen and F.M. Snell, eds. (New York: Academic Press), pp. 2–49.

Vokes, S.A., Ji, H., McCuine, S., Tenzen, T., Giles, S., Zhong, S., Longabaugh, W.J., Davidson, E.H., Wong, W.H., and McMahon, A.P. (2007). Genomic characterization of Gli-activator targets in sonic hedgehog-mediated neural patterning. Development *134*, 1977–1989.

Vokes, S.A., Ji, H., Wong, W.H., and McMahon, A.P. (2008). A genome-scale analysis of the cis-regulatory circuitry underlying sonic hedgehog-mediated patterning of the mammalian limb. Genes Dev. *22*, 2651–2663.

Waddington, C.H. (1942). Canalization of development and the inheritance of acquired characters. Nature *150*, 563–565.

Wartlick, O., Mumcu, P., Kicheva, A., Bittig, T., Seum, C., Julicher, F., and Gonzalez-Gaitan, M. (2011). Dynamics of Dpp signaling and proliferation control. Science *331*, 1154–1159.

Wolpert, L., Beddington, R., Brockes, J., Jessel, T., Lawrence, P., and Myerowitz, E. (1998). Organogenesis. In Principles of Development (London: Current Biology Publications), pp. 340–385.

Xu, M., Kirov, N., and Rushlow, C. (2005). Peak levels of BMP in the *Drosophila* embryo control target genes by a feed-forward mechanism. Development *132*, 1637–1647.

Yamada, T., Pfaff, S.L., Edlund, T., and Jessell, T.M. (1993). Control of cell pattern in the neural tube: motor neuron induction by diffusible factors from notochord and floor plate. Cell *73*, 673–686.

Zhou, Q., and Anderson, D.J. (2002). The bHLH transcription factors OLIG2 and OLIG1 couple neuronal and glial subtype specification. Cell *109*, 61–73.

EXTENDED EXPERIMENTAL PROCEDURES

Mouse Lines

Shh null allele was generated from a Cre-dependent conditional allele and genotyping was carried out as reported (Lewis et al., 2001). Genotyping for all other mouse lines was carried out as reported (Buscher et al., 1998; Lu et al., 2002; Schedl et al., 1996).

Tg(GBS-GFP) Reporter Transgenic Mice

8 concatemerized fragments of a FoxA2 enhancer that contains a Gli Binding Site (GBS; TTATGACGGAGGCTAACAAGCAGG GAACACCCAAGTAGAAGCTGGCTGTC) (Sasaki et al., 1997) were cloned upstream of the *hsp68* minimal promoter and *eGFP* in pBluescript SK+. To protect the activity of the *8GBS-hsp68-eGFP* from position effects when integrated into the mouse genome, this fragment was then subcloned in the BamH1 site of pJC13-1(Chung et al., 1993), which contains 2 insulators on each side of the BamH1 site. *Tg(GBS-GFP)* transgenic mice were generated by pro-nuclear injection into fertilized eggs from FVBN mice, and founder animals were genotyped for the *eGFP* sequence by PCR using the following primers: *5′TGCAGTGCTTCAGCCGCTAC3′; 5′CCAGCAGGACCATGTGATCG3′*. Two transient transgenic embryos at 70 hr postheadfold stage (hph) (e10.0) and 2 stable lines were obtained and all displayed similar patterns of eGFP expression (data not shown).

Immunohistochemistry and In Situ Hybridization

Mouse embryos from timed pregnant females were staged by counting somite number and fixed by immersion in 4% paraformaldehyde for 45min to 2hrs at 4°C. Fixed embryos were cryoprotected by equilibration in 15% sucrose, cryosectioned (14 μm) and processed for immunostaining or in situ hybridization (ISH). The following primary antibodies were used: rabbit anti-Olig2 (1:1000, Chemicon), sheep anti-GFP (1:1000, Biogenesis), rabbit anti-GFP (1:1000, Invitrogen), mouse against Nkx2.2 (1:25), rabbit anti-Ptch1 (Gift from Dr. Argraves [Morales et al., 2009]), guinea-pig anti-Gli2 (Gift from Dr Eggenschwiler [Ko et al., 2010]). Secondary antibodies were from Jackson Immuno Research: FITC donkey anti-sheep or donkey anti-rabbit IgG (H^+L); Cy5 donkey anti-goat IgG (H^+L) and Cy3 donkey anti-mouse, guinea-pig or rabbit IgG (H^+L). ISH was performed as described (Yamada et al., 1993), using as probes against mouse *Ptch1* (Ding et al., 1998), mouse *Gli1* and *GFP* (kindly provided by C.C. Hui and S. Gerety), respectively. Analyses were carried out using a Leica TCS SP2 and/or SP5 confocal microscope or a Zeiss Axioplan 2 and images processed with Adobe Photoshop CS4 software (Adobe Systems).

Chick In Ovo Electroporation

All chick misexpression constructs were based on pCAGGS expression vector (Niwa et al., 1991) engineered to bicistronically express nuclear targeted GFP. $Gli3A^{HIGH}$ (Stamataki et al., 2005), $Gli3A^{MED}$ (Stamataki et al., 2005), *SmoM2* (Hynes et al., 2000), *Olig2* (Novitch et al., 2001), and the two *Pax6-RNAi* (Das et al., 2006) constructs have been described previously. Hamburger and Hamilton (HH) stage 8-12 chick embryos were electroporated and incubated *in ovo* before dissecting and processing for immunohistochemistry.

Luciferase Assays

$Gli3A^{MED}$ (Stamataki et al., 2005), *Pax6-RNAi* (Das et al., 2006) constructs or pCAGGS as control were electroporated in chick embryos along with GBS-Luc, a firefly luciferase reporter construct containing eight repeats of the Gli binding site sequence (Sasaki et al., 1997) and a Renilla-luciferase reporter carrying the CMV immediate early enhancer promoter (Promega) for normalization. Embryos were homogenized with a douncer in Passive Lysis Buffer on ice and measurement of firefly and Renilla luciferase activities was performed using the Dual Luciferase Reporter Assay System (Promega).

Mouse Neural Plate Explant Culture

Neural plate tissue was isolated from 8-12hph mouse embryos and cultured as described in (Yamada et al., 1993). The medium for mouse explants was supplemented with N2 and B27 (GIBCO). For the GFP intensity quantification, at least five images containing approximately 150 cells each that had been randomly chosen from five to six explants were quantified with ImageJ (NIH) and the data were analyzed using MATLAB (Mathworks). Each experiment was performed independently more than once and gave reproducible results.

Embryo Culture Experiments

Mouse embryos from 8 to 12hph stages with intact yolk sacs, dissected from timed pregnant females, were cultured for 12h or 24h in medium (rat serum, Tyrode solution; 1:1). Cyclopamine (Sigma) dissolved in EtOH was used at a concentration of 10μM, while Purmorphamine (Calbiochem) dissolved in DMSO at a concentration of 5-10μM as indicated. Cultures were performed in a water-saturated roller-tube incubator at 37°C, 5% CO_2 and 20% O_2. After culture, embryos were fixed and processed. Gene expression patterns and the activity of the *Tg(GBS-GFP)* were always compared between embryos processed in the same culture experiment in appropriate control conditions.

Quantification of Protein Levels

Images were obtained using a Leica SP2 or SP5 confocal system. Stepwise bleaching was performed to test linearity of the GFP signal. Each image was the average of 3 optical sections, 0.2μm apart, taken from the middle of a 14μm cryosection. The fluorescence intensity of Nkx2.2, Olig2, GFP and Ptch1 expression was measured in rectangles of 16μm wide positioned from the ventral to dorsal midline along the apical side of the neural tube. Image J v.1.43 g image analysis software (NIH) was used. For comparison neural progenitor cells are on average ∼6μm wide in the dorsal-ventral axis and ∼12μm from apical to basal. Background measurements were obtained from mesoderm and/or the dorsal neural tube and these were subtracted from each assayed profile. To be able to compare the different sets of experiments, we normalized the values of GFP intensities in each set of experiments to those found in wild-type embryos (Figures 2B, S1D, and S1E). The positions of the Nkx2.2 and Olig2 expression domain boundaries were defined as the point at which fluorescent intensity reached 30% of the average of the fluorescence intensity within a domain, in which the fluorescence intensity was two folds higher than the background signal. The heat maps of GBS-GFP and Ptch1 intensity profiles along the D-V axis as well as the plots in Figure 1C, were generated from measures of mean fluorescence intensity after background subtraction in bins of 4μm. All the cell positions were recorded as the distance from the floor plate given as a % of the neural tube size. Data analysis and image generation were performed using Matlab (Mathworks, Natick, MA) or Excel (Microsoft Office). The heat maps provide a color-coded scale for individual datasets by associating the lowest value in the data matrix with a dark blue color and the highest value with the dark red color and interpolating linearly between these values.

Dynamical Systems Modeling

In the system of differential Equations 1–3, P, O and N represent the levels of Pax6, Olig2 and Nkx2.2 expression, respectively and G the levels of Shh-Gli signaling. The maximum rate of expression of each factor P, O, N is given by α, β, γ, respectively, and its degradation rate by k_i, (i = 1-3). Michaelis-Menten kinetics describes the induction of O and N by G. The cross-repressive interactions between the TFs are parameterized in the equations by Hill coefficients h_i, (i = 1-5) and critical values O_{critP}, N_{critP}, O_{critN}, P_{critN} and N_{critP}. In particular h_i influences the steepness of the sigmoidal curve of the repression function, which characterizes the change in expression of a given TF in response to its repressor. The higher the value of h_i, the steeper is the sigmoidal curve. The critical values dictate the value of the repressor for which the level of expression of the repressed TF is half the maximal level. Note that the dynamical system is designed to represent the epistatic relationships between the transcription factors and does not require the regulatory interactions to be direct.

The complexity of the system of differential Equations 1–3 precludes analytical solutions, thus the system was solved numerically using Matlab ode45 solver (Mathworks). The simulations give the P, O and N output values as a function of time for different values of the model parameters. For given values of G we calculated the values of P, O and N either prior to the system reaching equilibrium or once the system has reached a steady state. A full mathematical analysis of the system, including numerical analyses and a Heaviside simplification (in the limit where the Hill coefficients are infinitely large) that provides analytical solutions for the steady states and their stability, are described separately (J.P., K.M.P., and J.B., unpublished data).

We identified sets of model parameter values in which the system adopted the biologically relevant behavior: that is the levels of P, O, N switch from $P^{HIGH} \rightarrow O^{HIGH} \rightarrow N^{HIGH}$ states in response to progressively higher values of G (the parameters used throughout this study are shown in Table S2 and the states P^{HIGH}, O^{HIGH}, N^{HIGH} are defined as the condition in which the levels of P, O, N are above 1, respectively). A sensitivity analysis, halving or doubling each parameter value in turn, indicated that the parameters that describe the strength of the cross repression had a significant influence on the output of the system (Table S3). In the model, the repressive activity of the TFs is described by the combination of two independent sets of parameters: Hill coefficients and critical values. Stronger repressors are characterized by higher values of h_i and/or lower critical values. Keeping one set of parameters fixed, we determined the values of the other set that produced the relevant multistate switch. For both sets of parameters, the switch from O^{HIGH} to N^{HIGH} required N to inhibit O more strongly than O inhibited N (Tables S2 and S3). In addition, N had to strongly inhibit P, when O was removed from the system (β = 0).

For the extended system shown in Figure 7, the equations in C were solved numerically with the parameters given by: a = 3, b = 5, c = 5, d = 5, $h_{13} = 4$, $h_{14} = 6$, $h_{21} = 2$, $h_{23} = 1$, $h_{24} = 1$, $h_{31} = 6$, $h_{32} = 5$, $h_{34} = 1$, $h_{41} = 6$, $h_{42} = 5$, $h_{43} = 5$, $X_{2crit3} = 2$, $X_{2crit4} = 0.2$, $X_{3crit2} = 0.5$, $X_{3crit4} = 3$, $X_{jcriti} = 1$; [X_{jcriti} = critical value that describes the effect of j on i], $k_i = 1$; i = 1,2,3,4; M = 1).

SUPPLEMENTAL REFERENCES

Buscher, D., Grotewold, L., and Ruther, U. (1998). The XtJ allele generates a Gli3 fusion transcript. Mamm. Genome *9*, 676–678.

Chung, J.H., Whiteley, M., and Felsenfeld, G. (1993). A 5′ element of the chicken beta-globin domain serves as an insulator in human erythroid cells and protects against position effect in Drosophila. Cell *74*, 505–514.

Das, R.M., Van Hateren, N.J., Howell, G.R., Farrell, E.R., Bangs, F.K., Porteous, V.C., Manning, E.M., McGrew, M.J., Ohyama, K., Sacco, M.A., et al. (2006). A robust system for RNA interference in the chicken using a modified microRNA operon. Dev. Biol. *294*, 554–563.

Dessaud, E., Ribes, V., Balaskas, N., Yang, L.L., Pierani, A., Kicheva, A., Novitch, B.G., Briscoe, J., and Sasai, N. (2010). Dynamic assignment and maintenance of positional identity in the ventral neural tube by the morphogen sonic hedgehog. PLoS Biol. *8*, e1000382.

Dessaud, E., Yang, L.L., Hill, K., Cox, B., Ulloa, F., Ribeiro, A., Mynett, A., Novitch, B.G., and Briscoe, J. (2007). Interpretation of the sonic hedgehog morphogen gradient by a temporal adaptation mechanism. Nature *450*, 717–720.

Ding, Q., Motoyama, J., Gasca, S., Mo, R., Sasaki, H., Rossant, J., and Hui, C.C. (1998). Diminished Sonic hedgehog signaling and lack of floor plate differentiation in Gli2 mutant mice. Development *125*, 2533–2543.

Hynes, M., Ye, W., Wang, K., Stone, D., Murone, M., Sauvage, F., and Rosenthal, A. (2000). The seven-transmembrane receptor smoothened cell-autonomously induces multiple ventral cell types. Nat. Neurosci. *3*, 41–46.

Ko, H.W., Norman, R.X., Tran, J., Fuller, K.P., Fukuda, M., and Eggenschwiler, J.T. (2010). Broad-minded links cell cycle-related kinase to cilia assembly and hedgehog signal transduction. Dev. Cell *18*, 237–247.

Lewis, P.M., Dunn, M.P., McMahon, J.A., Logan, M., Martin, J.F., St-Jacques, B., and McMahon, A.P. (2001). Cholesterol modification of sonic hedgehog is required for long-range signaling activity and effective modulation of signaling by Ptc1. Cell *105*, 599–612.

Lu, Q.R., Sun, T., Zhu, Z., Ma, N., Garcia, M., Stiles, C.D., and Rowitch, D.H. (2002). Common developmental requirement for Olig function indicates a motor neuron/oligodendrocyte connection. Cell *109*, 75–86.

Morales, C.R., Fox, A., El-Alfy, M., Ni, X., and Argraves, W.S. (2009). Expression of Patched-1 and Smoothened in testicular meiotic and post-meiotic cells. Microsc. Res. Tech. *72*, 809–815.

Niwa, H., Yamamura, K., and Miyazaki, J. (1991). Efficient selection for highexpression transfectants with a novel eukaryotic vector. Gene *108*, 193–199.

Novitch, B.G., Chen, A.I., and Jessell, T.M. (2001). Coordinate regulation of motor neuron subtype identity and pan-neuronal properties by the bHLH repressor Olig2. Neuron *31*, 773–789.

Sasaki, H., Hui, C., Nakafuku, M., and Kondoh, H. (1997). A binding site for Gli proteins is essential for HNF-3beta floor plate enhancer activity in transgenics and can respond to Shh in vitro. Development *124*, 1313–1322.

Schedl, A., Ross, A., Lee, M., Engelkamp, D., Rashbass, P., van Heyningen, V., and Hastie, N.D. (1996). Influence of PAX6 gene dosage on development: overexpression causes severe eye abnormalities. Cell *86*, 71–82.

Stamataki, D., Ulloa, F., Tsoni, S.V., Mynett, A., and Briscoe, J. (2005). A gradient of Gli activity mediates graded Sonic Hedgehog signaling in the neural tube. Genes Dev. *19*, 626–641.

Yamada, T., Pfaff, S.L., Edlund, T., and Jessell, T.M. (1993). Control of cell pattern in the neural tube: motor neuron induction by diffusible factors from notochord and floor plate. Cell *73*, 673–686.

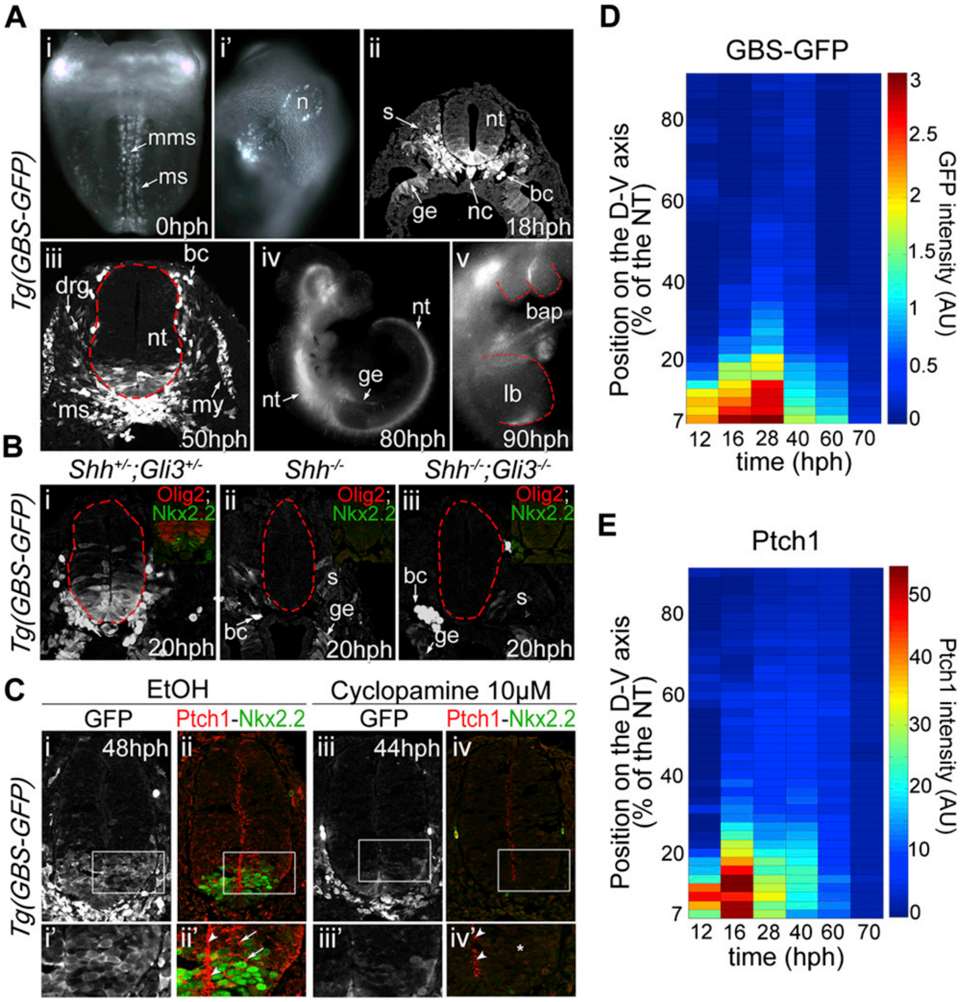

Figure S1. The Activity of the *Tg(GBS-GFP)* Reports Shh Signal Transduction, Related to Figure 1

(A) *Tg(GBS-GFP)* activity in Shh signaling responding tissues. The GFP expression was assessed in *Tg(GBS-GFP)* embryos at the indicated stages. i, i′, and iv, v are respectively ventral and lateral views of whole mount embryos. ii and iii are transverse sections at brachial levels. GFP was first observed in peripheral node cells (n), medial mesoderm (mms) as well as adjacent ventral mesoderm (ms). Later, GFP expression appeared throughout the ventral part of the neural tube (nt), the posterior limb bud (lb), the gut endoderm (ge), within the ventral somites (s) and then within the derived myotome (my), as well as within the first branchial pouch (bap). GFP was also present in blood cells (bc) and dorsal root ganglia (drg).

(B) The majority of *Tg(GBS-GFP)* activity is absent in embryos lacking Shh and positive Gli activity. Expression of GFP in *Tg(GBS-GFP)* in transverse sections of *Shh+/-;Gli3+/-* (i), *Shh-/-* (ii) and *Shh-/-;Gli3-/-* (iii) 20hph embryos at brachial levels.

(C) Inhibition of Smo activity by Cyclopamine decreases *Tg(GBS-GFP)* activity. Expression of GFP (white), Ptch1 (red) and Nkx2.2 (green) in *Tg(GBS-GFP)* mouse embryos cultured from the 12hph stage for 24h in control media (i–ii′) or with the Smo inhibitor Cyclopamine (iii–iv′). i′, ii′, iii′, iv′ are expanded images of the regions indicated with white boxes in i, ii, iii, iv, respectively. Arrowheads indicate puncta of Ptch1 protein at the apical surface of neuroprogenitor cells, while arrows point out Ptch1 protein in vesicle-like structures at more basal levels. The star in iv′ indicates the reduced levels of Ptch1 protein at basal levels upon Cyclopamine treatment.

(D) Quantification of *Tg(GBS-GFP)* activity in the neural tube. The heat map indicates GFP intensity (mean in arbitrary units (AU)) along the dorsal-ventral axis (distance from the apical side of the floor plate cells in % of the neural tube).

(E) Quantification of Ptch1 protein expression along the dorsal-ventral axis of the neural tube. The heat maps indicate Ptch1 intensity (mean in arbitrary units (AU)) at the indicated dorsal-ventral positions.

For embryo stages see Table S1.

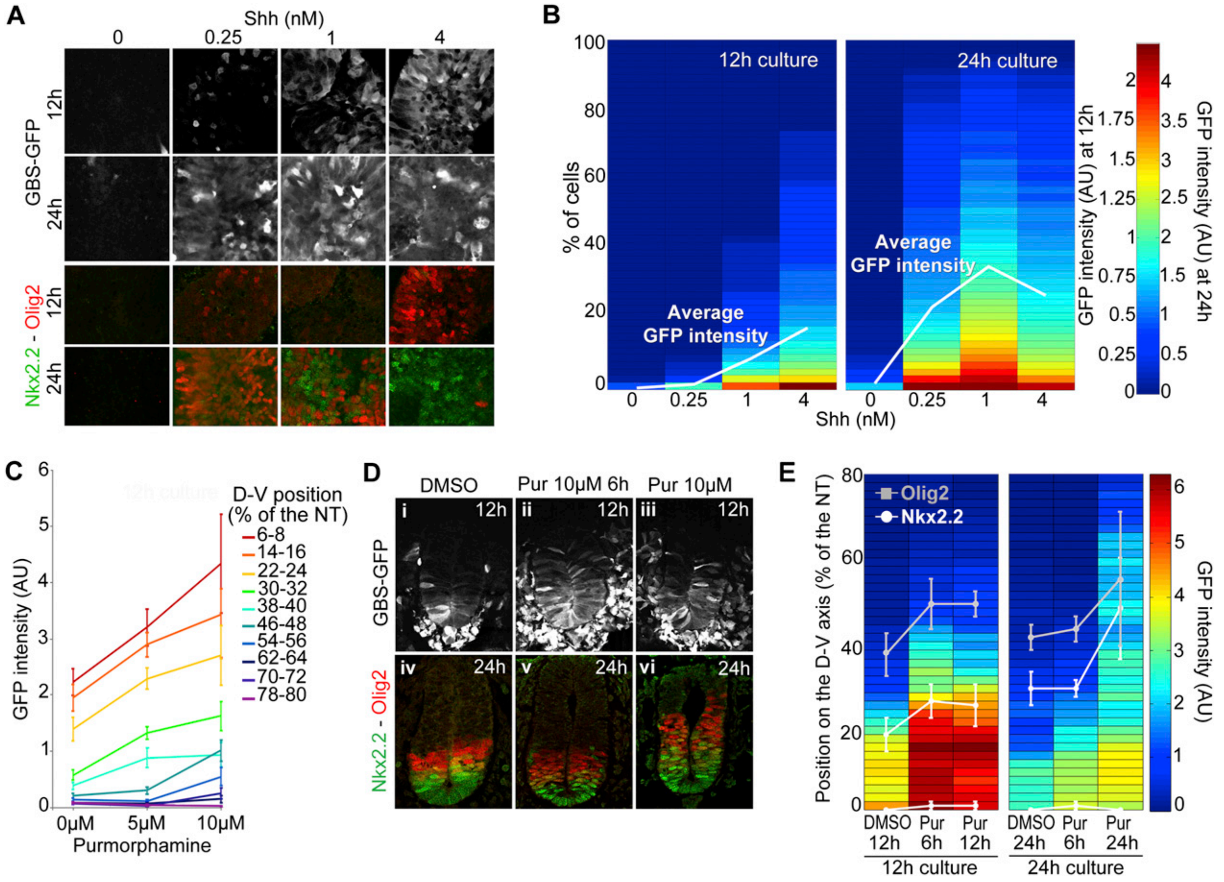

Figure S2. Tg(GBSGFP) Responds to Changes in Shh Signaling, Related to Figure 1

(A and B) Levels of *Tg(GBS-GFP)* activity track levels of Shh signaling. (A) Naïve [i] explants extracted from the intermediate neural plate of 8-12hph *Tg(GBS-GFP)* mouse embryos and cultured in presence of the indicated Shh concentrations for 12 or 24h, analyzed for the expression of GFP (white), Olig2 (red) and Nkx2.2 (green). Similarly to *ex vivo* experiments performed with chick tissue (Dessaud et al., 2007; 2010), increasing concentration and duration of Shh exposure led to a progressive switch from Olig2 to Nkx2.2 induction in mouse explants. (B) Quantification of *Tg(GBS-GFP)* activity in explants treated with the indicated Shh concentrations for 12h and 24h. The intensity of GFP in each cell of the assayed region was measured (arbitrary unit (AU); n ≥ 3 explants/condition) and plotted as a % of cells within explants. The white line reports the mean GFP intensity calculated from these measurements. Exposure to different concentrations of Shh for 12h resulted in corresponding differences in both the number of cells containing detectable *Tg(GBS-GFP)* activity and the levels of GFP within individual cells. At 24h, consistent with the induction of feedback mechanisms in cells responding to Shh that changes the sensitivity of cells to Shh over time, the level of GFP in explants was no longer perfectly correlated with Shh concentration.

(C) The activity of *Tg(GBS-GFP)* responds to increases in the levels of Smo activity *in vivo*. Quantification of *Tg(GBS-GFP)* activity in embryos cultured *ex vivo* with 0, 5 and 10μM of Purmorphamine, a Smo agonist, for 12h from stage 8-12hph. Average GFP intensity (mean ± s.e.m. in arbitrary unit (AU)) at relative distances (% of the neural tube) from the floor plate.

(D and E) The establishment of the Nkx2.2 dorsal boundary is robust to transient increases in Shh signaling. (D) GFP (white), Nkx2.2 (green) and Olig2 (red) expression in *Tg(GBS-GFP)* embryos after 12h or 24h of culture *ex vivo* from stage 8-12hph. Embryos were exposed to 0μM or 10μM Purmorphamine for 6h (Pur 10μM 6h) or for the entire period of culture (Pur 10μM).

(E) Quantification of *Tg(GBS-GFP)* activity in embryos after 12h or 24h of culture *ex vivo* and exposed to 0μM or 10μM Purmorphamine for 6h (Pur 6h) or for the entire period of culture (Pur 12h or Pur 24h). Heat maps illustrate GFP intensity (mean in arbitrary units (AU)) along the dorso-ventral axis (distance from the floor plate in μm) and the positions of the dorsal boundary of Nkx2.2 (white line) and Olig2 (gray line) expression (mean ± s.d.) in embryos from the indicated conditions (n ≥ 3 embryos/condition). In embryos cultured for 6h in presence of 10μM of Purmorphamine, the levels of Gli activity detected after 12h of culture were as much increased as in embryos exposed with the agonist for 12h. In contrast at 24h, while the position of Nkx2.2 and Olig2 dorsal boundaries in embryos exposed to the drug for 6h was similar to that of DMSO treated embryos, these boundaries were shifted dorsally in embryos treated for 24h with Purmorphamine.

For embryo stages see Table S1.

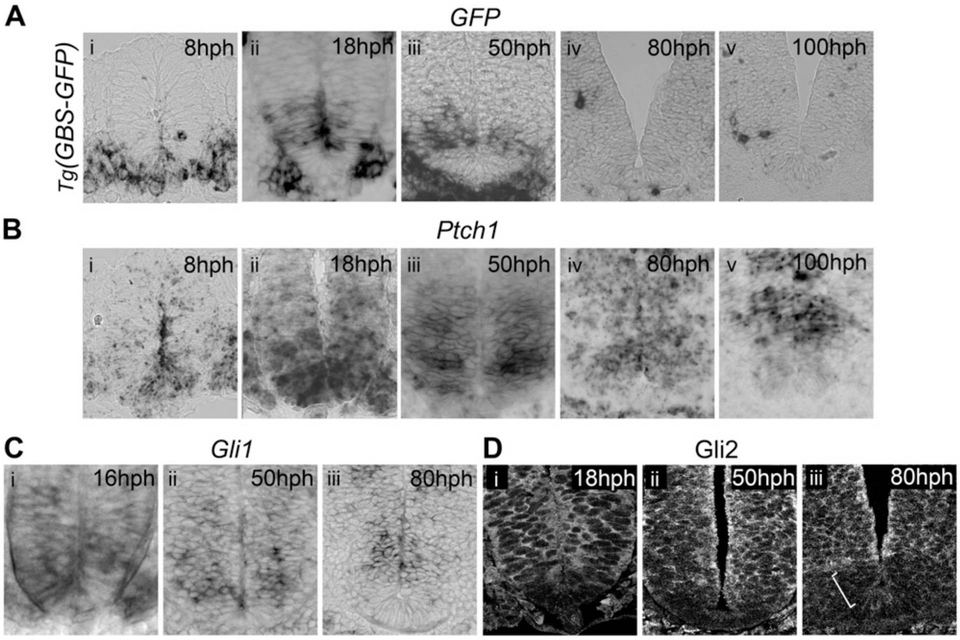

Figure S3. Correlation between the Dynamics of *Tg(GBS-GFP)* Activity and *Ptch1*, *Gli1*, and Gli2 Expression, Related to Figure 1

Temporal-spatial profiles of *GFP* (A), *Ptch1* (B) and *Gli1* (C) mRNA and Gli2 protein (D) at brachial levels of *Tg(GBS-GFP)* (A) and wild type embryos (B–D) at the indicated stages.

(A) *GFP* transcripts were detected by 8hph in the ventral neural tube (i). From this stage, the expression of *Tg(GBS-GFP)* expanded progressively dorsally (ii and iii). Concomitantly, *Tg(GBS-GFP)* expression was extinguished within the most ventral cells of the neural tube which represent the presumptive floor plate (ii and iii). After 50hph *GFP* mRNA could not be detected within neural progenitor cells (iv and v; data not shown).

(B) *Ptch1* mRNA was present in the ventral neural tube by 8hph (i). Between 10-18hph, *Ptch1* expanded dorsally and was distributed in a graded manner; strong in ventral midline cells and weaker dorsally (ii). Then, its expression was progressively extinguished from the floor plate, but it remained expressed at moderate levels within the p3 domain and at low levels within the dorsal half of the neural tube (iii and iv). By 100hph, *Ptch1* transcripts were detected in most neural progenitor cells, with the exception of the floor plate. The levels of its expression were relatively weak within the p3 domain and stronger within the pMN domain (v).

(C) *Gli1* mRNA initially displays a ventral to dorsal gradient that extends to half the size of the neural tube (i), its expression is then progressively downregulated from the floor plate cells (ii) and later from the p3 cells (iii).

(D) Gli2 protein is detected within the entire neural tube (data not shown) but gets progressively downregulated from the floor plate cells (I and ii) and the p3 domain (white brackets) (iii). For embryo stages see Table S1.

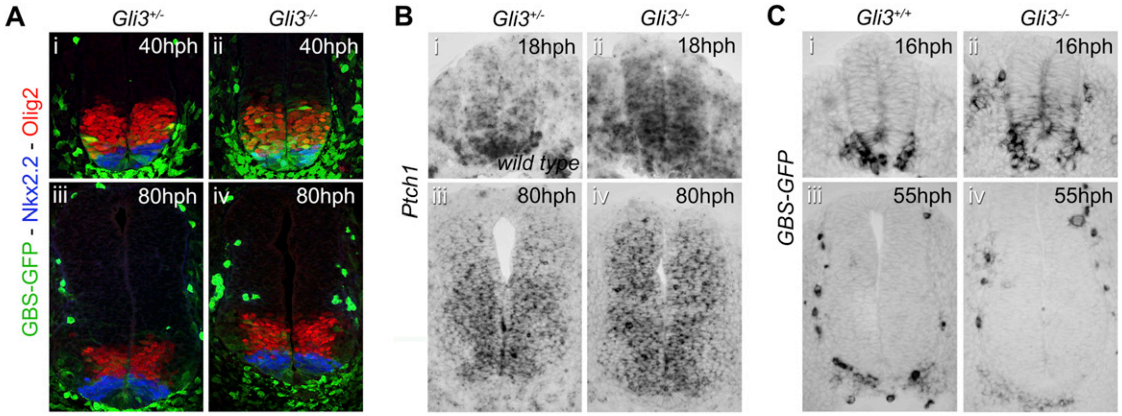

Figure S4. Comparison of Gli Activity with Expression of Nkx2.2 and Olig2 in Mutants for *Gli3*, Related to Figure 2

(A) Expression of Nkx2.2 (blue), Olig2 (red) and GFP (green) in forelimb regions of mouse embryos of the indicated genotype and stage. The amplitude and range of GFP was markedly increased in 40hph *Gli3*-/- embryos compared to *Gli3*+/- littermates of the same stage, whereas the expression of Nkx2.2 and Olig2 was similar in mutant and control embryos (i and ii). Expression of Nkx2.2, Olig2 and GFP was comparable in *Gli3*+/- and *Gli3*-/- mouse embryos at 80hph (iii and iv).

(B) *Ptch1* expression in forelimb regions of wild type, *Gli3*+/- and *Gli3*-/- embryos at the indicated stages. Compared to wild type, a dorsal expansion and increase in the intensity of *Ptch1* expression was observed in 18hph and 80hph *Gli3*-/- embryos.

(C) *In situ* hybridization for *GFP* in *Tg(GBS-GFP)* embryos lacking or wild-type for Gli3 at the indicated stages. Similarly to the profile of GFP protein expression, the *GFP* transcripts were dorsally expanded in *Gli3*-/- mutants compared to wild type embryos (i and ii). This ectopic expression of *GFP* lasted for a short period of time, as *GFP* was not detected in wild type nor *Gli3* mutant embryos by 55hph (iii and iv).

A summary of embryo staging is given in Table S1.

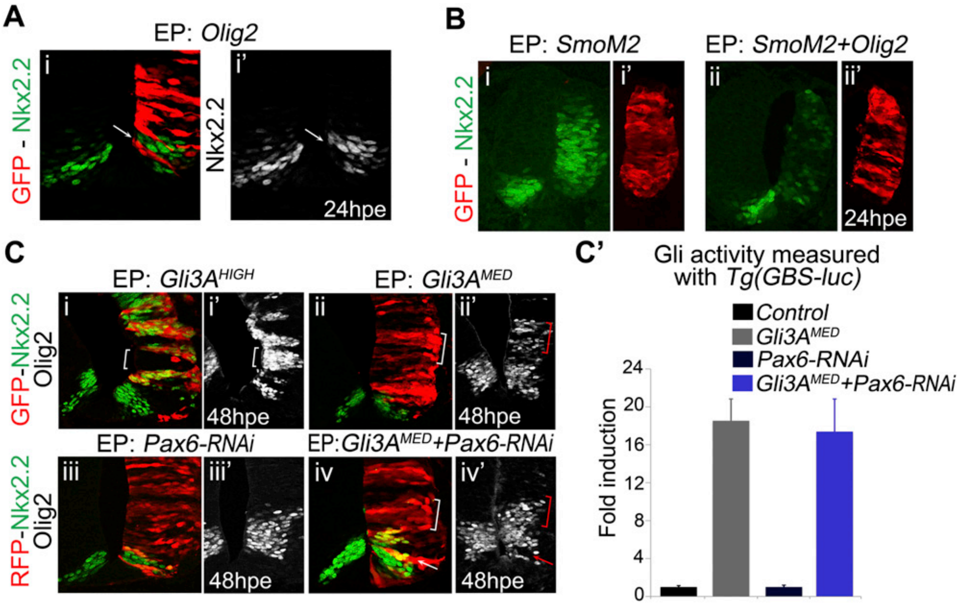

Figure S5. Nkx2.2 Is Repressed by Olig2 and Induced by Medium Levels of Gli Activity in Absence of Pax6, Related to Figure 3

(A and B) Olig2 represses Nkx2.2. HH12 stage chick *in ovo* electroporation of *Olig2* (A) or *SmoM2* with (Bii and Bii′) or without *Olig2* (Bi and Bi′) expression constructs. Embryos were assayed for Nkx2.2 expression (green in Ai, B, white in Ai′) 24h post electroporation (hpe). Olig2 was sufficient to repress Nkx2.2 in a cell-autonomous manner (arrows in A). Expression of Olig2 blocks the ability of SmoM2, a constitutively active form of Smoothened, to up-regulate Nkx2.2 (B). (C and C′) Nkx2.2 responds to medium levels of Gli activity after Pax6 knock-down. (C) Electroporation of the indicated constructs at HH12 stage chick embryos and analysis of Nkx2.2 (green) and Olig2 (white) expression at 48hpe. All sections are from the forelimb and anterior thoracic regions of embryos. Nkx2.2 and Olig2 were ectopically induced in dorsal and intermediate regions of neural tube, electroporated with *Gli3AHIGH* (i and i′). Olig2 (ii′), but not Nkx2.2 (ii) were induced in *Gli3AMED*-transfected cells. Two *Pax6-RNAi* constructs were used to knock-down Pax6 expression. When introduced alone into the neural tube these had no effect on Nkx2.2 (iii) or Olig2 (iii′) expression. Transfection of *Gli3AMED* and *Pax6-RNAi* together resulted in the dorsal expansion of Nkx2.2 (iv) and Olig2 (iv′; red brackets). This indicates that reducing Pax6 expression potentiates the ability of moderate levels of Gli activity to induce Nkx2.2. Moreover, these embryos exhibit repression of Olig2 expression probably as a result of Nkx2.2 dorsal expansion (arrows in iv and iv′). (C′) HH10-12 stage chick embryos were electroporated with the indicated constructs together with the GBS-Luc reporter and normalization plasmid. Graphs show the relative luciferase activity 24h after transfection (mean ± s.e.m.). Reduction in the level of Pax6 by RNAi did not alter the level of Gli activity induced by *Gli3AMED*.

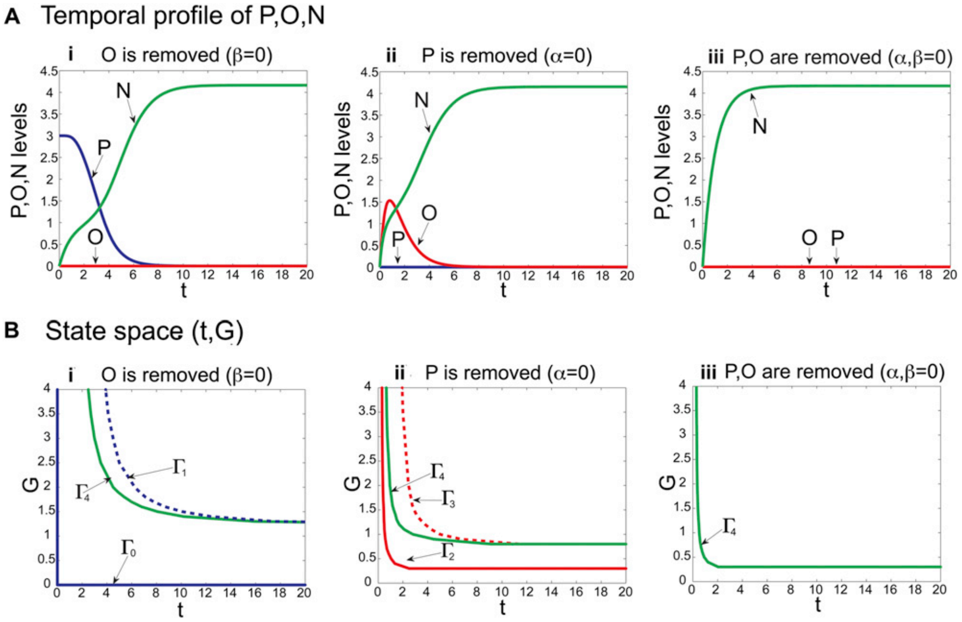

Figure S6. The Temporal Behavior of the Gene Regulatory Network, Related to Figure 4

(A) Temporal profiles of P (blue), O (red) and N (green) when: (i) O is removed (β=0); (ii) P is removed (α=0); (iii) both P and O are removed (α, β=0) (See also Figure 4C). Each dataset was generated by solving the model equations numerically with Matlab (ode45 solver) for the model parameters given in Table S2 and G=5.

(B) (t,G) State space representations of the model output indicating the values of G and t for which P^{HIGH}, O^{HIGH}, or N^{HIGH} dominate. The lines (Γ1-Γ4) indicate the values of G and t at which P (blue, Γ0, Γ1), O (red, Γ2, Γ3), N (green, Γ4) are equal to 1; solid lines indicate the threshold at which the value increases above 1, in the positive t or G direction; dotted lines indicate the threshold at which the value decreases below 1, in the positive t or G direction. The line Γ0 represents the expression of P when t, G=0. Removing O from the model (β=0) shifts the threshold of N domination to lower values of G and shorter periods of time (i). Removal of P (α=0) from the model decreases more markedly the value of G and time at which N is induced (ii). The threshold for N domination shifts further to lower values of G and time when both P and O are removed from the model (α, β=0) (iii).

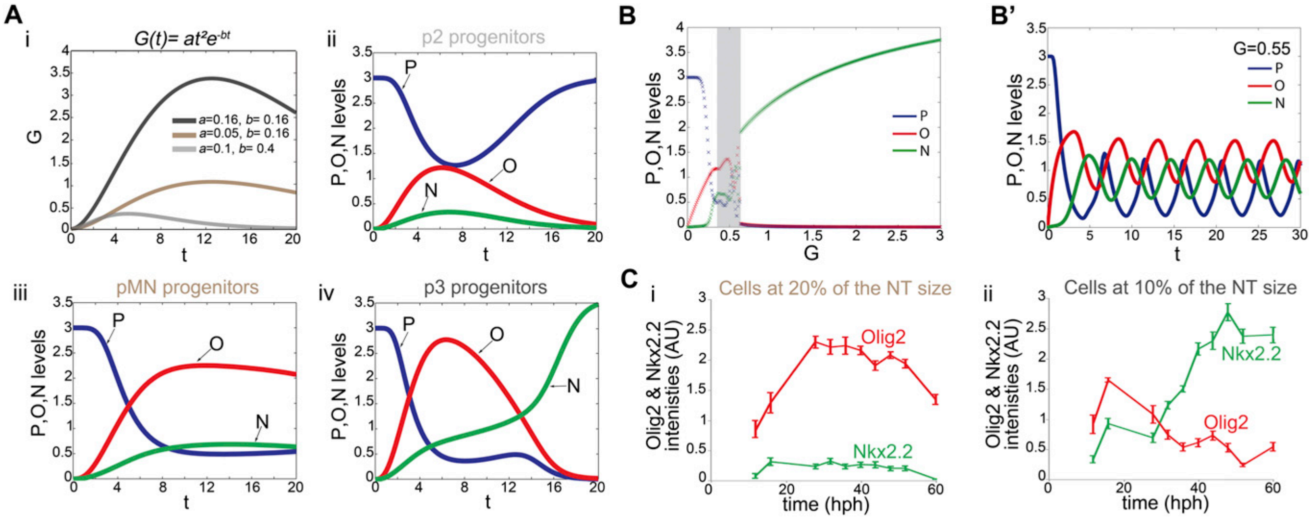

Figure S7. The GRN Has the Potential to Generate Oscillations and to Interpret a Temporally Changing Level of Signaling, Related to Figure 7

(A) To simulate, in the model, the temporally changing Gli activity profile observed *in vivo* (Figures 1D and 1E), the function $G(t) = at^2e^{-bt}$, was used. Three profiles of *G(t)* (represented with black, brown and gray lines) were generated using different values of *a* and *b* (i). Numerically derived profiles of P (blue), O (red) and N (green) as a function of time for the three simulated values of *G(t).* For the high *G(t)* profile (black line) P decreases and O is induced at early times, then N increases and O decreases. This behavior mimics the profile of gene expression observed in the p3 domain (iv). For the medium *G(t)* profile (brown line), O increases and P decreases to a low but sustained level. This mimics the gene expression profile in pMN progenitors (iii). For the low *G(t)* profile (gray line), the value of G never reaches a level sufficient to activate O and P is expressed throughout the simulation – this mimics the establishment of the p2 domain (ii).

(B and B′) Changing the parameters of the model (Table S2) reveals the potential for oscillations. For a range of G values (shading gray area) (B) the system produced oscillations in time (t) (B′). The periodic output was evident within the switch from O^{HIGH} to N^{HIGH} for intermediate values of G (G=0.75). Parameter values α=3, β=5, γ=5, *h1*=6, *h*2=10, *h*3=5, *h4*=1, *h5*=2, *k1*=*k2*=*k3*=1, *NcritP*=*OcritP*=1, *PcritN*=0.5, *OcritN*=5, *NcritO*=1.

(C) Quantification of Nkx2.2 and Olig2 expression levels over time in cells located at 10% (ii) and 20% (ii) of the NT size. Within cells committed to a pMN fate at 20% of the NT size, the levels of Olig2 expression progressively increase to reach a plateau around ~25hph. In these cells Nkx2.2 levels remain barely detectable. In contrast in cells fated to be p3 cells, Olig2 expression levels peak at 18hph then progressively decrease. The decrease in Olig2 expression correlated with the induction of Nkx2.2, the expression of which plateaus at ~50hph. These dynamics predicted by the model in Aiii and Aiv are strikingly reminiscent of that of Ci and Cii, respectively.
